# Anastellin impacts on the processing of extracellular matrix fibronectin and stimulates release of cytokines from coronary artery smooth muscle cells

**DOI:** 10.1038/s41598-022-26359-9

**Published:** 2022-12-21

**Authors:** Jianfei He, Jonas Hyld Steffen, Peter Waaben Thulstrup, Jannik Nedergaard Pedersen, Max B. Sauerland, Daniel E. Otzen, Clare L. Hawkins, Pontus Gourdon, Michael J. Davies, Per Hägglund

**Affiliations:** 1 Department of Biomedical Sciences, University of Copenhagen, Copenhagen, Denmark; 2 Department of Chemistry, University of Copenhagen, Copenhagen, Denmark; 3 Interdisciplinary Nanoscience Center (iNANO), Department of Molecular Biology and Genetics, Aarhus University, Aarhus, Denmark; 4 Present Address: Arla Foods Ingredients Group P/S, Sønderupvej 26, 6920 Videbæk, Denmark

**Keywords:** Biochemistry, Biophysics, Cell biology

## Abstract

Anastellin, a recombinant protein fragment from the first type III module of fibronectin, mimics a partially unfolded intermediate implicated in the assembly of fibronectin fibrils. Anastellin influences the structure of fibronectin and initiates in vitro fibrillation, yielding “superfibronectin”, a polymer with enhanced cell-adhesive properties. This ability is absent in an anastellin double mutant, L37AY40A. Here we demonstrate that both wild-type and L37AY40A anastellin affect fibronectin processing within the extracellular matrix (ECM) of smooth muscle cells. Fibronectin fibrils are diminished in the ECM from cells treated with anastellin, but are partially rescued by supplementation with plasma fibronectin in cell media. Proteomic analyses reveal that anastellin also impacts on the processing of other ECM proteins, with increased collagen and decreased laminin detected in media from cells exposed to wild-type anastellin. Moreover, both anastellin forms stimulate release of inflammatory cytokines, including interleukin 6. At the molecular level, L37AY40A does not exhibit major perturbations of structural features relative to wild-type anastellin, though the mutant showed differences in heparin binding characteristics. These findings indicate that wild-type and L37AY40A anastellin share similar molecular features but elicit slightly different, but partially overlapping, responses in smooth muscle cells resulting in altered secretion of cytokines and proteins involved in ECM processing.

## Introduction

Fibronectin (FN) is a major extracellular matrix (ECM) protein implicated in the regulation of cell adhesion, migration, differentiation, and proliferation. “Cell-derived” FN released from endothelial cells, smooth muscle cells and fibroblasts is assembled into insoluble, elastic fibrils that interact with other ECM proteins (e.g. collagens and proteoglycans) and integrins on cell surfaces^1,2^. On the other hand, soluble plasma FN synthesized by hepatocytes is released into the circulation and recruited to sites of injury where it plays a key role in blood clot formation^3^. Plasma FN is also deposited into ECM fibrils together with locally-synthesized, cell-derived FN^4–6^. The mechanism of FN polymerization in the ECM is not fully established, but there is strong evidence suggesting that cytoskeletal actins exert a pulling force on FN through interactions via membrane-bound integrin receptors, which expose hidden (“cryptic”) sites that stabilize interactions between individual protomers, thereby promoting polymerization^2,7,8^.

FN is a multimodular glycoprotein consisting of two subunits connected by two disulfide bonds near the C-termini^9^. Each subunit comprises 12 type I modules (FNI), 2 type II modules (FNII) and 15–17 type III modules (FNIII), that are organized into functional domains that enable macromolecule binding. Two of the FNIII modules (EDA and EDB) are uniquely expressed in cell-derived FN. Anastellin (AN), a small protein fragment derived from the C-terminal part of the first type III module of FN (FNIII_1_), initiates polymerization of plasma FN in vitro, resulting in the formation of ‘superfibronectin’, a polymer which resembles ECM-derived FN fibrils at the microscopic level. In addition to its influence on FN fibrillation, AN also inhibits cell proliferation, has anti-angiogenic properties, and suppresses tumor growth and metastasis^10–12^. AN also interacts with heparin-based polysaccharides^13^. These interactions are probably mediated by a cluster of positively-charged cryptic residues that are important for FN-cell interactions in the native FNIII_1_ module^14^.

Besides its ability to initiate FN polymerization in vitro, AN also modulates the structure of ECM-incorporated FN, and impacts on cell behavior^15,16^. With fibroblasts, AN activates p38 mitogen-activated protein kinase (MAPK) signaling with subsequent effects on cytoskeletal organization and cell cycle progression^15,16^. AN also modulates vascular endothelial growth factor (VEGF) signaling in endothelial cells, and inhibits lysophospholipid signaling via Ras/ERK^17,18^. Furthermore, AN stimulates the release of inflammatory cytokines from fibroblasts and mononuclear cells through activation of NF-κB dependent pathways^19,20^.

The structural features of AN that are responsible for these activities are incompletely resolved, but it has been demonstrated that mutation of Leu37 and Tyr40 to Ala (i.e. an L37AY40A mutant) removes the capacity of AN to induce FN polymerization^21^. However, the L37AY40A mutant retains the ability of wild-type (wt) AN to modulate FN structure in assembled ECM, and impact on MAPK signaling^22^. We therefore hypothesized that the activity of AN to induce plasma FN polymerization, and remodeling of FN in established ECM, operate through different mechanisms. We also hypothesized that AN may induce alterations in the processing of other extracellular proteins. This was examined using a proteomics approach to quantify the levels of ECM-related proteins secreted from human coronary artery smooth muscle cells (HCASMC) exposed to either wt or L37AY40A AN. In addition, we investigated the molecular features of the two forms of AN, and compared their interactions with isolated plasma FN and heparin, with the aim to provide new insights into the mechanisms of AN-mediated FN fibrillation and ECM remodeling.

## Results

### Impact of AN on the structure of FN fibrils generated by HCASMC

**Figure 1:**
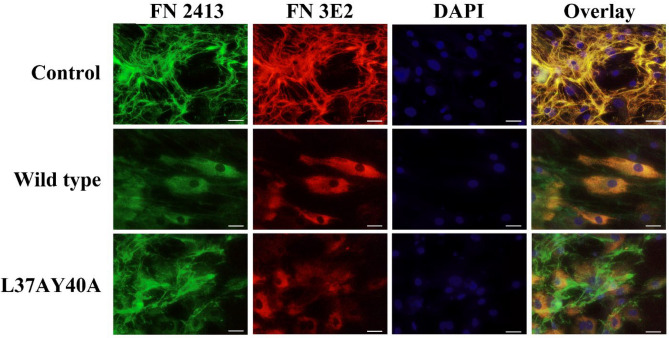
AN influences FN structure in ECM from cells cultured in growth media. Primary HCASMC at an initial density of 2.25 × 10^4^ cells per well in 8-well chamber slides were cultured overnight in growth media and then incubated in growth media containing 30 μM AN (wt or L37AY40A) for 48 h. The cells were then fixed and permeabilized followed by incubation with primary antibodies at 4 °C overnight. Total FN was visualized by an anti-FN pAb (2413, 1:500, green channel) and a mAb against the EDA module of cell-derived FN (3E2, 1:500, red channel). Nuclei were counterstained using DAPI (blue channel). Overlays of the three channels are shown on the right. Scale bars: 20 μm. Pixel intensity plots from three independent experiments are displayed in Supplementary Fig. S1.

The influence of wt and L37AY40A AN on the assembly of the ECM laid down by HCASMC cultures was examined by seeding cells on chamber culture slides and incubating these in growth media without or with added AN. The resulting cultures were then probed for FN by immunofluorescence microscopy. A marked decrease in the extent of extracellular FN fibrils was observed for cells exposed to wt AN for 48 h, as detected by use of both a polyclonal FN antibody (FN 2413), and a monoclonal (FN 3E2) antibody directed against the EDA module of cell-derived FN (Fig. 1). In contrast, the FN detected in the cells treated with AN appears to be localized within the cell, or at the cell surface. With L37AY40A AN, a decrease in the levels of FN fibrils was  also observed with the FN 3E2 mAb, which only detects FN containing the EDA module, whereas significant incorporation of FN into fibrils was using the pAb which recognizes both cell-derived FN species and plasma FN (Fig. 1).

A similar trend was observed when the HCASMC cultures were decellularized, to leave cell-free ECM, before incubation with the FN antibodies. Higher levels of FN in the ECM were detected from the cells incubated with the L37AY40A mutant compared to wt AN (Supplementary Fig. S2). These different effects of wt and L37AY40A AN imply that the L37 and Y40 residues of AN are important determinants for the observed effects on the structure of cell-derived FN. In contrast to the cell microscopy images (Fig. 1), staining of fibrillar FN was detected with the 3E2 antibody in the decellularized samples derived from cells exposed to AN, albeit at a lower level relative to control samples (Supplementary Fig. S2). This discrepancy may be explained by increased epitope accessibility when cells growing on top of the deposited matrix are removed.

To determine whether the FN incorporated into the ECM fibrils was associated with AN, decellularized ECM from cells exposed to AN was probed for both FN (using the pAb) and AN (using an antibody against the His-tag on the recombinant protein). The resulting data showed strong co-localization of FN with either wt or L37AY40A AN in the ECM, consistent with specific interactions between the two proteins (Supplementary Fig. S3a). The extent of AN incorporation was dependent on the concentration of AN added, and whether this was wt or L37AY40A AN, with the latter being incorporated to a greater extent than the former at modest, but not high AN concentrations (Supplementary Fig. S3b).

### FN fibrils in cells exposed to AN are restored in the presence of plasma fibronectin

**Figure 2:**
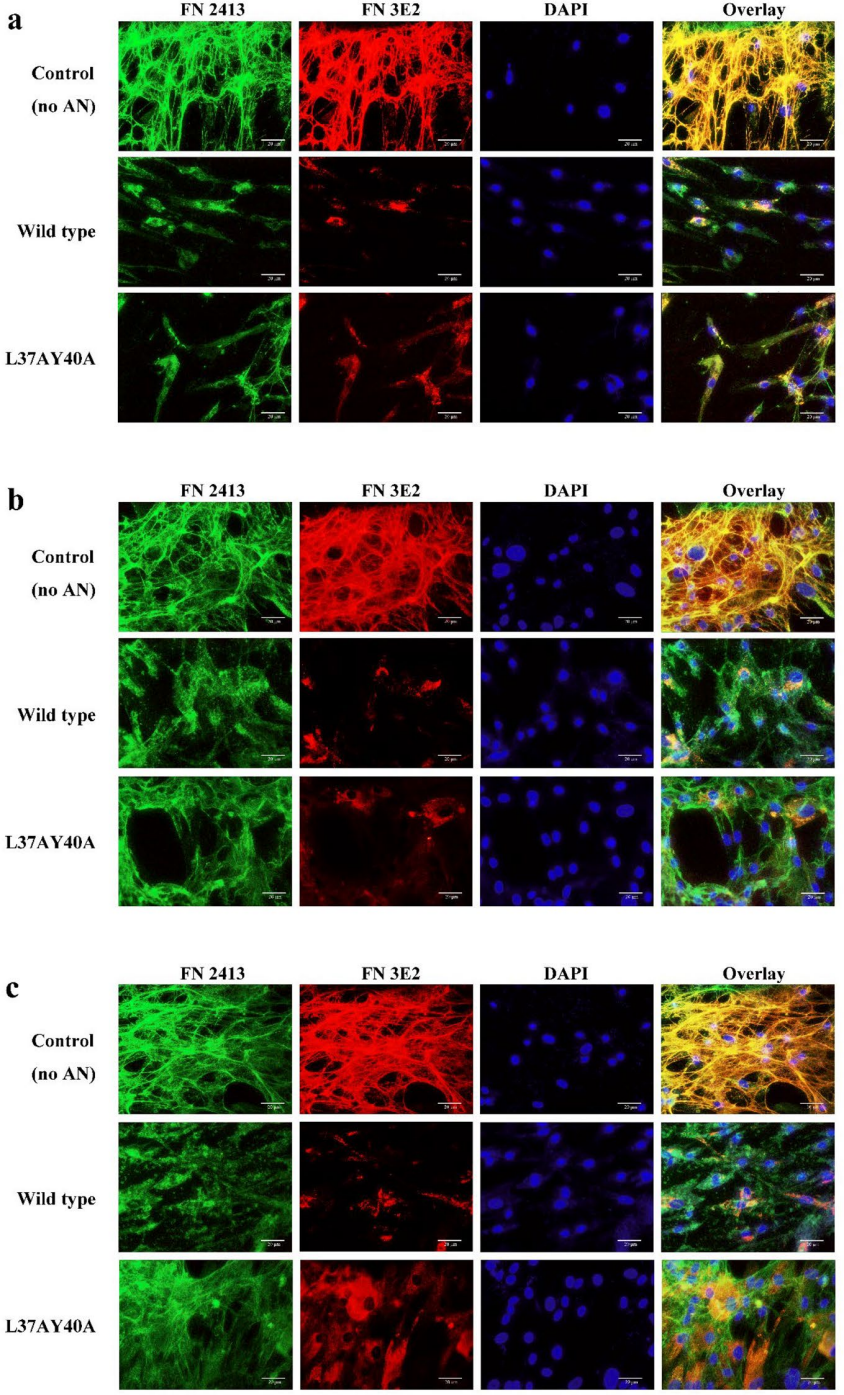
AN influences FN structure in ECM from cells cultured in basal media. Primary HCASMC at an initial density of 2.25 × 10^4^ cells per well in 8-well chamber slides were cultured in basal media overnight and then incubated in basal media supplemented with 30 μM AN (wt or L37AY40A) and either 0 (**a**), 10 (**b**) or 100 (**c**) ug mL^−1^ FN for 24 h. HCASMCs on the slides were then fixed and permeabilized followed by incubation with primary antibodies at 4 °C overnight. Total FN was visualized by anti-FN pAb (2413, 1:500, green channel) and cell-derived FN detected using a mAb that recognizes the EDA epitope (3E2, 1:500, red channel); nuclei were counterstained using DAPI (blue channel). Overlays of images are shown on the right. Scale bars: 20 μm. Pixel intensity plots from three independent experiments are displayed in Supplementary Fig. S2.

To examine the influence of the cell media on the extracellular FN fibrils, HCASMC were subjected to serum starvation, then incubated for 24 h with wt or L37AY40A AN in serum-free basal media. With both wt and L37AY40A AN, most of the FN detected with either the pAb or mAb appears to be mainly cell associated, and levels of fibrillar FN in the ECM were diminished relative to the control cells without AN (Fig. 2a). These findings indicate that the abundance of FN fibrils in cells exposed to L37AY40A AN is dependent on plasma-derived FN present in the growth (but not basal) media, or another component present in this media. In order to test this hypothesis, cells were incubated in basal media supplemented with different levels of plasma FN. These additions yielded significantly higher levels of fibrillar FN in cells exposed to AN (Fig. 2b,c vs 2a). The plasma FN-dependent increase in FN fibrils is most obvious for cells exposed to L37AY40A AN.

Cells were also incubated in basal media for a shorter incubation time (2 h) and investigated by immunofluorescence microscopy. In contrast to the longer incubation time of 24 h (Fig. 2a), the differences in levels of fibrillar FN detected in the ECM from cells incubated in the absence or presence of AN were less pronounced after 2 h (Supplementary Fig. S5). These observations suggest that AN may influence the turnover of FN fibrils in the ECM from HCASMC.

### AN modulates intracellular levels of FN to a moderate extent

To examine whether AN modulates the synthesis and processing of FN, HCASMC lysates were prepared from cells that had been cultured in growth media containing wt or L37AY40A AN for 48 h. Separation of the proteins present in the cell lysates (prepared using deoxycholate) by SDS-PAGE and subsequent immunoblotting using the FN pAb demonstrated that decreased cellular levels of FN were detected in cells exposed to either wt or L37AY40A AN, and there were no statistically-significant differences between the levels in the cells exposed to the two proteins (Supplementary Fig. S6a–c). In addition, quantitative real-time PCR (qPCR) analysis revealed that the levels of the FN-encoding transcript FN1 were not significantly different between the cells exposed to wt or L37AY40A AN for 2 h (Supplementary Fig. S6d). The modest changes at the transcriptional level suggest that the lack of extracellular FN fibrils in cells exposed to AN is not mainly due to suppression of FN expression.

### AN induces modest alterations in cellular metabolic activity, viability and proliferation

To determine if some of the above differences in FN processing and matrix incorporation arise from other AN-mediated effects on the cells, cell viability was assayed by quantifying lactate dehydrogenase (LDH) release from the cells, relative to the total amount in the cells and media; these values showed no significant difference (Supplementary Fig. S7a). Furthermore, the mitochondrial reductive capacity (metabolic activity) of HCASMC co-incubated with wt or L37AY40A AN, at 15 or 30 μM, for 48 h was examined using the MTS assay. No significant changes, when compared to controls without AN, were detected with either wt or L37AY40A AN at 15 μM, though a small, but significant, enhancement was observed with wt AN, but not L37AY40A AN, at 30 μM (Supplementary Fig. S7b). However, at both concentrations, a significant increase in metabolic activity was detected for wt compared to L37AY40A AN. The total cell numbers (determined by trypan blue exclusion) did not show any significant decrease with either AN form, with a modest increase (relative to control cells) detected with wt AN at both concentrations (Supplementary Fig. S7c). No significant differences were detected between the two forms of AN. qPCR analysis of the mitosis-related genes PCNA (Proliferating Cell Nuclear Antigen), CCNA1 (Cyclin-A1) and CCNB1 (Cyclin-B1) indicated no significant differences in the expression of these genes between cells exposed to AN (wt or L37AY40A) and control cells incubated in the absence of AN (Supplementary Fig. S8). Together these data indicate that the changes in FN processing and matrix incorporation do not result from AN-mediated toxicity or major alterations in cell metabolic activity or proliferation.

### AN stimulates release of cytokines and proteins involved in ECM processing

Culture supernatants from HCASMC were subjected to proteomics analysis by LC–MS/MS to evaluate if AN (wt or L37AY40A) induced changes in the levels of specific proteins secreted from the cells. ECM-associated proteins that are present at higher levels in conditioned media from cells exposed to wt and L37AY40A AN, relative to controls without AN exposure, are listed in Table 1 and include multiple collagens (I, III, IV, V, VI), enzymes involved in ECM processing (MMP2) as well as protease inhibitors (e.g. tissue factor pathway inhibitor 2). Some ECM-related proteins were more abundant in the conditioned media from cells exposed to wt AN, but not L37AY40A AN (e.g. cathepsin D and Z) or vice versa (e.g. peroxidasin and thrombospondin). Several cytokines, including interleukin 6 (IL-6) and CXC chemokines 1, 3, 5 and 6, showed the greatest increase in abundance in the cell conditioned media from cells exposed to wt or L37AY40A AN, relative to controls (Supplementary Fig. S9). In addition, CXC chemokines 2 and 10 were detected in supernatants from cells exposed to wt and L37AY40A AN, but not in supernatants from control cells (Supplementary Data S2). The level of IL-6 in the supernatants was also assessed by immunoblotting, with a dose-dependent increase detected from cells exposed to L37AY40A AN (Supplementary Fig. S10). Table 2 lists ECM-related proteins present at lower levels in the cell conditioned media from cells exposed to AN, with these including annexin, fillagrin, nidogen and versican. Some ECM proteins such as laminin and FN were detected at lower levels in cells exposed to wt AN, but not L37AY40A AN, relative to the controls.

**Table 1:** ECM-associated proteins present at higher levels in conditioned media from HCASMC exposed to wt or L37AY40A AN compared to cells without AN exposure.

Protein	Gene	Uniprot	wt	L37AY40A
72 kDa type IV collagenase	MMP2	P08253	X	X
Alpha-2-macroglobulin	A2M	P01023		X
Antithrombin-III	SERPINC1	P01008	X	
Cathepsin D	CTSD	P07339	X	
Cathepsin Z	CTSZ	Q9UBR2	X	
CCN family member 2	CCN2	P29279	X	X
Coiled-coil domain-containing protein 80	CCDC80	Q76M96	X	
Collagen alpha-1(I) chain	COL1A1	P02452	X	X
Collagen alpha-1(III) chain	COL3A1	P02461	X	X
Collagen alpha-1(VI) chain	COL6A1	P12109	X	X
Collagen alpha-2(I) chain	COL1A2	P08123	X	X
Collagen alpha-2(IV) chain	COL4A2	P08572	X	X
Collagen alpha-2(V) chain	COL5A2	P05997	X	X
Collagen alpha-2(VI) chain	COL6A2	P12110	X	X
Collagen alpha-3(VI) chain	COL6A3	P12111		X
C-X-C motif chemokine 1	CXCL1	P09341	X	X
C-X-C motif chemokine 3	CXCL3	P19876	X	X
C-X-C motif chemokine 5	CXCL5	P42830	X	X
C-X-C motif chemokine 6	CXCL6	P80162	X	X
Cystatin-B	CSTB	P04080		X
Cystatin-C	CST3	P01034	X	X
EGF-containing fibulin-like extracellular matrix protein 1	EFEMP1	Q12805	X	X
Extracellular matrix protein 1	ECM1	Q16610	X	X
Fibrillin-1	FBN1	P35555		X
Glia-derived nexin	SERPINE2	P07093	X	X
Inactive serine protease PAMR1	PAMR1	Q6UXH9		X
Inhibin beta A chain	INHBA	P08476		X
Insulin-like growth factor-binding protein 6	IGFBP6	P24592	X	X
Insulin-like growth factor-binding protein 7	IGFBP7	Q16270	X	X
Interleukin-6	IL6	P05231	X	X
Lysosomal protective protein	CTSA	P10619	X	
Metalloproteinase inhibitor 1	TIMP1	P01033	X	X
Metalloproteinase inhibitor 2	TIMP2	P16035	X	X
Pappalysin-1	PAPPA	Q13219	X	X
Peroxidasin homolog	PXDN	Q92626		X
Pigment epithelium-derived factor	SERPINF1	P36955	X	X
Plasma protease C1 inhibitor	SERPING1	P05155	X	X
Procollagen C-endopeptidase enhancer 1	PCOLCE	Q15113	X	X
Procollagen-lysine,2-oxoglutarate 5-dioxygenase 1	PLOD1	Q02809		X
Procollagen-lysine,2-oxoglutarate 5-dioxygenase 2	PLOD2	O00469		X
Profilin-1	PFN1	P07737	X	
Serpin B6	SERPINB6	P35237		X
SPARC	SPARC	P09486	X	X
Stromelysin-1	MMP3	P08254		X
Thrombospondin-1	THBS1	P07996		X
Thrombospondin-2	THBS2	P35442		X
Tissue factor pathway inhibitor 2	TFPI2	P48307	X	X

A full list of all regulated proteins is available in Supplementary Data S1.

**Table 2:** ECM-associated proteins present at lower levels in conditioned media from HCASMC exposed to wt or L37AY40A AN compared to cells not exposed to AN.

Protein	Gene	Uniprot id	wt	L37AY40A
Agrin	AGRN	O00468	X	
Annexin A1	ANXA1	P04083	X	X
Annexin A2	ANXA2	P07355	X	X
Annexin A5	ANXA5	P08758	X	X
Annexin A6	ANXA6	P08133	X	X
Antileukoproteinase	SLPI	P03973	X	
Collagen alpha-1(XII) chain	COL12A1	Q99715		X
Deleted in malignant brain tumors 1 protein	DMBT1	Q9UGM3	X	
Fibronectin	FN1	P02751	X	
Filaggrin-2	FLG2	Q5D862	X	X
Filamin-B	FLNB	O75369	X	
Galectin-1	LGALS1	P09382	X	
Inter-alpha-trypsin inhibitor heavy chain H2	ITIH2	P19823	X	
Inter-alpha-trypsin inhibitor heavy chain H3	ITIH3	Q06033		X
Laminin subunit alpha-4	LAMA4	Q16363	X	
Laminin subunit beta-1	LAMB1	P07942	X	
Laminin subunit gamma-1	LAMC1	P11047	X	
Nidogen-2	NID2	Q14112	X	X
Protein S100-A8	S100A8	P05109	X	
Protein-glutamine gamma-glutamyltransferase K	TGM1	P22735		X
Serpin B12	SERPINB12	Q96P63	X	
Versican core protein	VCAN	P13611	X	X
Vimentin	VIM	P08670	X	

A full list of all regulated proteins is available in Supplementary Data S1.

### The capacity of AN to modulate FN cell adhesiveness is not directly linked to superfibronectin formation

Incubation of plasma FN with wt AN, and subsequent coating onto plates, before addition of cells, resulted in an increase in adhesion of HCASMC, as detected by the use of cells preloaded with the fluorescent dye calcein-AM (Supplementary Fig. S11a). No significant differences were detected between the effects of wt or L37AY40A AN on cell adhesion. Formation of fibrillar superfibronectin by wt but not L37AY40A AN, was confirmed by turbidity measurements and sedimentation assays (Supplementary Fig. S12) indicating that superfibronectin formation and cell adhesion are distinct and separate events. Control experiments with AN alone (i.e. no FN) showed that neither form of AN exhibited direct cell adhesiveness (Supplementary Fig. S11a). In contrast, when the plasma FN was pre-coated on plates and then treated with AN, before addition of cells, a significant decrease in adhesion was detected with L37AY40A but not wt AN, when compared to controls (Supplementary Fig. S11b). These data suggest that L37AY40A AN may adversely affect the adhesiveness of FN by blocking interaction sites or inducing conformational changes in the material on the plates, with this being dependent on L37 and/or Y40.

### Wt and L37AY40A AN induce conformational changes in plasma FN

**Figure 3:**
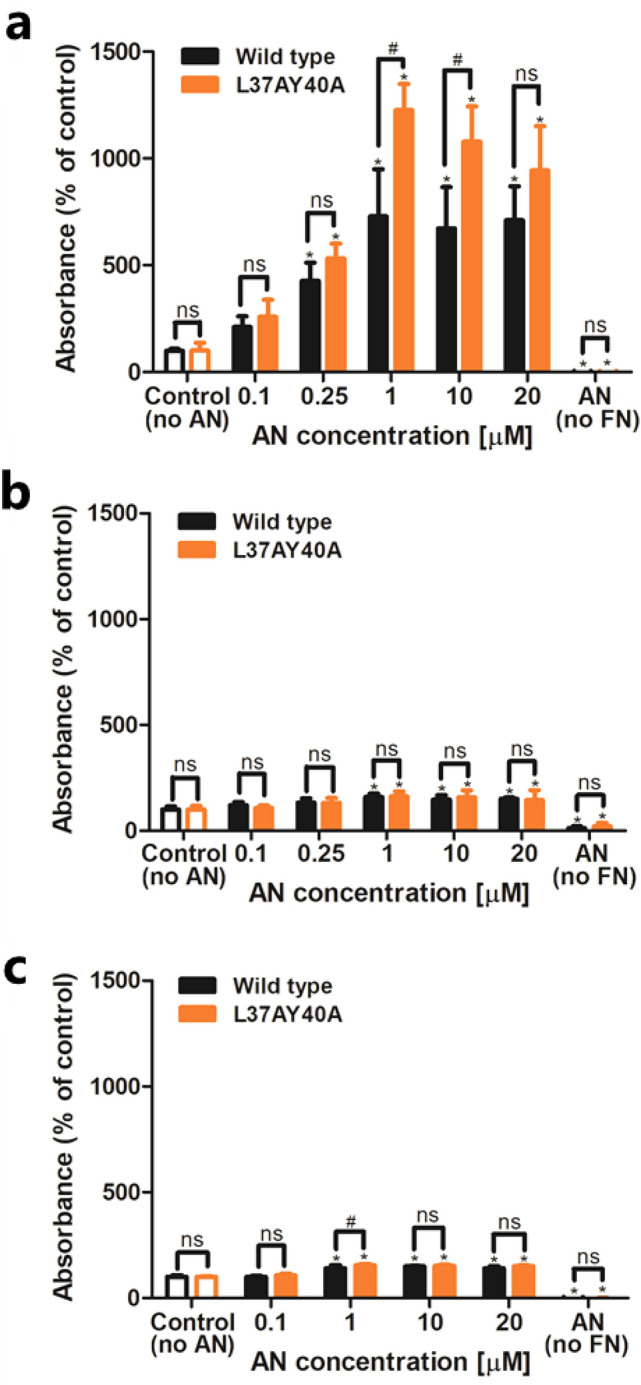
AN induces a conformational switch in plasma FN. Wt or L37AY40A AN were mixed with FN (1 μg mL^−1^) in a 96-well plate overnight at 21 °C then probed in an ELISA format using: (**a**) a mAb that recognizes the heparin-2 binding domain of FN (A32, 1:500), or (**b**) a pAb against FN (2413, 1:500). Alternatively, (**c**) plasma FN (20 μg mL^−1^) was pre-coated on plates, then incubated with wt or L37AY40A AN overnight, and probed by the A32 mAb. For controls without FN 20 μM AN was used. Data are presented as means ± SD from three independent experiments, analyzed by 2-way ANOVA with Tukey's multiple comparison test. * indicates significant difference from the control without AN at the p < 0.05 level. # indicates significant difference between the two AN forms at the p < 0.05 level. ‘ns’ indicates an absence of significant differences.

The structural conformation of plasma FN exposed to wt and L37AY40A AN was probed in ELISA assays with mAb A32 that recognizes a conformation-sensitive epitope in the heparin 2 domain of FN^23^. Incubation of plasma FN in solution with increasing concentrations of AN gave a dose-dependent enhancement of antibody recognition, with a maximal signal detected at ≥ 1 μM AN (Fig. 3a). A similar trend was observed with L37AY40A AN, although higher values were detected with the latter at 1–10 μM when compared to wt AN (Fig. 3a). Corresponding assays performed with pAb 2413 gave only marginal increases in the ELISA signal with increasing AN concentrations (Fig. 3b), with these small increases possibly arising from recognition of AN by this pAb (Fig. 3b). Similar experiments performed with wt or L37AY40A AN added to FN pre-coated on plates, also yielded a significantly increased signal from mAb A32, although the magnitude of these changes was less than for the solution experiments (Figs. 3a vs 3c). These data suggest that both wt and L37AY40A AN induce conformational changes in plasma FN, with these changes being greater for FN in solution, than pre-coated on plates. The binding of AN to FN (either pre-coated or co-incubated) under these conditions was confirmed by an antibody that recognizes the His-tag of AN. These experiments showed a dose-dependent increase in AN binding to FN with a maximal signal detected at ≥ 1 μM AN (Supplementary Fig. S13). Thus, the increased recognition by mAb A32 correlates with AN binding to FN.

The observed increase in recognition of the heparin-binding 2 domain in FN prompted investigation of a possible increase in affinity of heparin for FN exposed to AN. When wt or L37AY40A AN was co-incubated with FN overnight, an increase in binding of fluorescein-tagged heparin was observed relative to a AN control without FN, but not relative to a FN control without AN (Supplementary Fig. S14a). Similar results were observed when AN was added to pre-coated FN, except that no significant increase was detected for wt AN relative to control without FN (Supplementary Fig. S14b).

### Wt and L37AY40A AN display similar overall molecular structures but different heparin binding profiles

**Figure 4:**
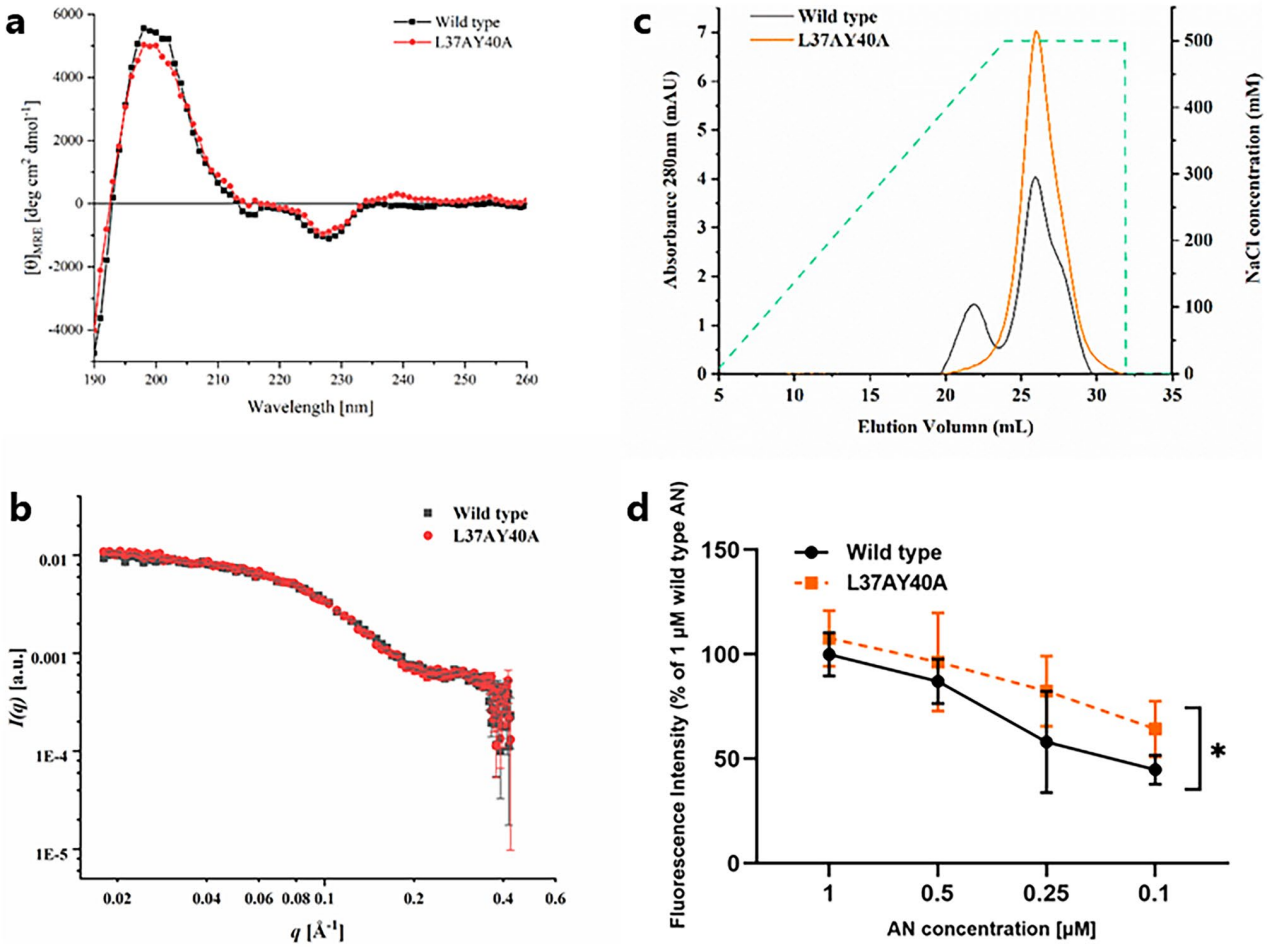
Molecular features of wt and L37AY40A AN. (**a**) Far-UV CD spectroscopy (190–260 nm) of wt and L37AY40A AN (0.1 mg mL^−1^) at 25 °C in 1 mm cells; for further details see Experimental procedues. (**b**) SAXS data for wt and L37AY40A AN (100 μM, 0.93 mg mL^−1^) measured in 10 mM phosphate buffer, pH 7.4, on an optimized NanoSTAR SAXS instrument. Data are displayed as a function of the scattering vector, q, with a scaling factor used to compare the data for wt and L37AY40A AN. (**c**) Separation of wt and L37AY40A AN on a Hitrap Heparin HP column. Samples were applied in 20 mM sodium phosphate buffer, pH 7.4, and eluted with a gradient of 0–500 mM NaCl in 20 mM phosphate buffer at 1 mL min^−1^. (**d**) Binding of fluorescein-tagged heparin (4 mg mL^−1^) to AN (wt or L37AY40A) coated on plates at 21 °C overnight, detected by fluorescence measurements (λ_ex_ 495 nm and λ_em_ 525 nm). Data are presented as means ± SD from three independent experiments, analyzed by paired-sample T-tests. *Indicates statistically significant differences between wt and L37AY40A AN at the p < 0.05 level.

The influence of molecular structure on the observed effects of wt and L37AY40A AN was investigated by far-UV circular dichroism (CD) spectroscopy (Fig. 4a). Similar spectra were detected for both forms indicating that the L37A and Y40A mutations do not perturb the beta-sheet structure of the protein. Small-angle X-ray scattering (SAXS) analyses yielded radii of gyration (*R*g) that were not significantly different (wt AN 18.2 ± 0.2 Å, L37AY40A AN 18.6 ± 0.1 Å), consistent with a similar size and molecular conformation (Fig. 4b). It is therefore concluded that the differences between wt and L37AY40A AN outlined above are not due to major perturbations in overall structure.

To examine other potential differences which might rationalize the observed alterations in functionality of L37AY40A AN, the heparin-binding profiles of wt and mutant AN were examined by chromatographic separation on a heparin-binding column (Fig. 4c). L37AY40A AN eluted as a single peak at high NaCl concentrations, whereas wt AN eluted as two peaks with one of these having a similar elution time to L37AY40A AN (Fig. 4c). These data suggest that wt AN binds to the heparin column in two different conformations, with one of these not adopted by L37AY40A. Isolation of the species that give rise to the two peaks with wt AN, and subsequent re-chromatography of the separate fractions, resulted again in the detection of two forms (data not shown) suggesting that the two wt AN conformations exist in a dynamic equilibrium. The heparin affinity of AN was also examined using a fluorescein-labelled heparin analogue in a binding assay format. Under these conditions a significant increase in heparin binding affinity was observed for L37AY40A AN (Fig. 4d), with the amount of bound label increasing with the AN concentration.

## Discussion

The data obtained in this study demonstrate that AN has a major impact on the structure of FN in the ECM derived from HCASMC cultures, with a marked decrease in the extent of extracellular FN fibrils observed for cells exposed to this protein. FN fibrils in the presence of the inactive L37AY40A AN mutant were restored by supplementation of the basal cell media with plasma FN, which suggests that these events are independent of superfibronectin formation. These results are interesting in relation to previous data reporting that the antiangiogenic activity of AN in vivo is dependent on the presence of plasma FN^24^. Earlier work has reported different effects of AN on FN assembly and turnover with this being dependent on the experimental conditions and cell type. Thus, Bordoulous et al. observed that AN promoted disassembly of FN fibrils generated by fibroblast and endothelial cells^15^, whereas Klein et al. showed that AN induces a conformational switch in FN that decreases recognition of an epitope in the EDA module^16^. We also observed a diminished signal from the EDA module in assembled ECM, but no conclusive evidence was obtained for a conformational change of FN in the ECM, since a loss of signal from fibrillar FN was also observed with the pAb detecting total FN. Previous studies demonstrated that conformational changes in FN induced by AN alters cell signaling via the VEGF and Ras/ERK pathways^17,18^. Such alternations have been associated with the anti-proliferative effects of AN on endothelial and vascular smooth muscle cells^10^. In contrast, our data are consistent with an absence of significant changes in cell viability and proliferation, and a slight increase in metabolic activity observed on exposure of HCASMC to wt AN.

Since FN plays a central role as a scaffold and “master regulator” in the ECM, we reasoned that the observed gross changes in the morphology of FN fibrils would also be associated with changes in overall ECM composition. Proteomics analysis of conditioned media from cells exposed to wt and L37AY40A AN revealed strikingly different protein profiles relative to control cells incubated without AN. Some ECM proteins such as collagens were detected at higher levels in supernatants from cells exposed to either wt or L37AY40A AN. This may be due to inhibition of collagen fibril assembly or an increase in collagen turnover. In contrast, lower levels of both FN and laminin were detected from cells exposed to wt, but not L37AY40A AN. These results do not agree with the lower intracellular levels of FN detected in cells exposed to both wt and L37AY40A AN. This may be due to differences in the secretory or extracellular processing pathways for wt and L37AY40A AN. Peroxidasin was detected at higher levels in the conditioned media from cells exposed to L37AY40A AN, but not wt AN. Peroxidasin plays a key role in the intermolecular cross-linking of the NC1 domains of collagen, and increased levels of this enzyme may therefore influence ECM structure and particularly the extent of matrix crosslinking^25^. Several of the identified proteins are known to interact directly with FN, including alpha-2-macroglobulin, CCN2 and fibrillin^26–28^. Wt and L37AY40A AN also stimulated the release of proteases (e.g. collagenase and pappalysin) and protease inhibitors (e.g. TIMP1-2 and antithrombin) that influence ECM turnover. Some of these proteases (e.g. MMP2) are known to cleave FN, and may thus catalyze turnover of FN fibrils in the ECM^29^. Several of the upregulated proteins, including tissue factor pathway inhibitor 2 and thrombospondin (elevated only in L37AY40A AN) display antiangiogenic properties^30,31^. These observations may be of relevance to the antiangiogenic and antimetastatic properties of AN observed in mice with human tumor cell grafts^11^. Release of inflammatory cytokines such as IL-6 and CXC chemokines 3, 5 and 6 was also stimulated in cells exposed to wt and L37AY40A AN. IL-6 is known to be released from vascular smooth muscle cells after exposure to bacterial antigens, different pro-inflammatory molecules or mechanical stress, and has been associated with TLR4-dependent signaling^32,33^. A similar inflammatory response has previously been reported for fibroblasts and mononuclear cells exposed to AN^19,20^.

In addition to its ability to modulate ECM assembly, AN also promotes FN polymerization in vitro in the absence of cells. The resulting polymer is often referred to as “superfibronectin” on the assumption that “superadhesiveness” correlates with FN fibrillation^34^. We demonstrate here that AN-induced enhancement of FN adhesiveness is, at least partially, independent of FN polymerization, as no significant difference was observed between wt and L37AY40A AN that lacks the ability to form superfibronectin. An increase in AN-mediated cell adhesiveness was only seen when AN (wt or L37AY40A) was co-incubated with FN in solution; when AN was added to FN coated on plates decreased adhesiveness was seen. These data suggest that the increase in cell adhesiveness is mediated by direct interactions between AN and FN in solution, and that AN added to FN coated on plates may either block cell access to adhesion sites, or that the FN incorporated into ECM cannot undergo marked structural changes.

Our data also demonstrate that interactions of AN with plasma FN exposes an epitope in the heparin-2 binding domain. These alterations are detected at concentrations of AN where no superfibronectin formation is observed (i.e. < 1 μM). Furthermore, the exposure of the heparin-2 binding domain occurs primarily when AN and FN are co-incubated in solution, and not when AN is added to pre-coated FN. These observations suggest that the exposure of the epitope is due to conformational changes rather than polymerization of FN and that these changes occur primarily with the soluble, non-polymerized protein.

The structure of both soluble and fibrillar FN is dynamic and is influenced by interactions with various ligands^35–39^. Whether these functional alternations are dependent on large-scale conformational changes or more subtle structural rearrangements is unclear^34,40–42^. Previous data indicates that AN interacts with the FNIII_1,2,3,11_ modules^46–48^, but there are no reports of direct interactions between AN and the heparin-2 binding domain in FN that is localized in FNIII_12-14_. It is however possible that AN—FNIII_1,2,3,11_ interactions induce long-range conformational changes that enhance exposure of the heparin-2 binding domain. This process may drive superfibronectin formation, as previous data has demonstrated that the heparin-2 binding domain interacts with FNIII modules and plays a key role in FN assembly^49^. On the other hand, it is also possible that exposure of the heparin-2 binding domain contributes to the observed inhibition of FN matrix assembly in cells exposed to AN; similar effects have been demonstrated with cells exposed to an isolated heparin-2 binding domain fragment^49^.

The folded structure of the FNIII module that AN is derived from, is found in many proteins^50^. Even though FNIII modules display high sequence diversity, the FNIII fold is highly conserved and is composed of two anti-parallel beta sheets, comprised of 3 (A, B, E) and 4 (C, D, F, G) strands respectively. AN lacks the two first beta strands (A, B) of the FNIII fold, and is instead composed of a single beta sheet (C, D, F, G), with the remaining (E) strand present as a flexible loop containing L37 and Y40^21^. Stine et al. have reported that AN can engage in beta-strand exchange with FN, whereby the two first beta-strands from FNIII_3_ (A, B) form a beta-sheet with the E strand from AN^46^. It is therefore speculated that L37 and Y40 may stabilize contacts between the beta-strands of AN and FN and that these contacts are disrupted in the L37AY40A mutant and impact on fibril formation and matrix assembly of FN. Our data indicate that wt and L37AY40A AN have a similar molecular structure. It is therefore likely that the absence of fibrillation activity of L37AY40A results from local perturbations around the mutated residues, altered hydrophobicity, or disruption of hydrogen bonds involving the hydroxyl group of Y40. In this context it is interesting to reflect on the differences between wt and L37AY40A AN in terms of heparin binding. AN contains three clustered positively-charged residues in a _13_RWRPK_17_ motif that binds negatively-charged heparin polysaccharides^14^. These residues constitute a “cryptic” heparin-binding motif that is exposed when FN is subjected to chemomechanical forces that partially unfold the FNIII_1_ module, or as a result of proteolytic processing. In AN, the _13_RWRP_16_ portion of this motif is located on beta-strand C, while K17 is positioned in the loop between strands C and D. The distance between Y40 and R13, the closest neighbors between L37/Y40 and the _13_RWRPK_17_ motif is ~ 14 Å, which rules out direct contacts. It therefore seems likely that the differences in heparin binding are due to changes in the local vicinity of the _13_RWRPK_17_ motif.

In conclusion, the current data demonstrates that AN has a strong influence on the processing of FN in HCASMC and elicits release of inflammatory cytokines and proteins involved in ECM turnover. Further investigations are required to determine the detailed mechanisms responsible for these observations, but the data reported here indicate that L37 and Y40, play a key role. These findings may be of therapeutic importance, particularly in regard to wound healing, angiogenesis and cell dispersal in tumor metastasis, where FN-dependent matrices are of key importance.

## Experimental procedures

### Materials

All chemicals, including lyophilized human plasma FN (F1056) and anti-FN monoclonal antibody (mAb) 3E2, were purchased from Sigma-Aldrich (St Louis, Missouri, USA), unless stated otherwise. All solutions were prepared with Milli-Q grade water (Millipore Advantage A10; Merck-Millipore, Billerica, MA, USA). Anti-FN pAb (2413), anti-His tag mAb (18,184) and anti-IL 6 pAB (6672) were from Abcam. Anti-FN heparin-2 binding fragment mAb (A32) was from Life Technologies. Anti-β actin mAb (8929) was from R&D System. Horseradish peroxidase (HRP)-conjugated sheep anti-mouse/anti-rabbit whole immunoglobulin (IgG) secondary antibodies were purchased from Millipore. DAPI (4′,6-diamidino-2-phenylindole) and Alexa Fluor 488-conjugated anti-rabbit and 594-conjugated anti-mouse secondary antibodies were from Molecular Probes (Thermo Fisher Scientific). Fluorescein-conjugated heparin (~ 18 kDa; H7482) and RIPA Lysis Buffer (89,900) was purchased from Thermo Fisher Scientific. Recombinant AN was produced in *Escherichia coli* essentially as described previously^51^. The L37AY40A mutant of AN (translated sequence in Supplementary Fig. S15) was prepared using the QuickChange Lightning Site-Directed Mutagenesis kit (Agilent, USA) according to the manufacturer’s instructions. Primers used for engineering the mutant was purchased from TAG Copenhagen A/S, Denmark. The sequences of the primers are 5’-ACGCGAACAGCGCGACCATTAAGGGTCTGAAACCGGG-3’ (forward) and 5’-TCGCGCTGTTCGCGTGACCCGGGATGGTCGC-3’ (reverse). Correct mutation was verified by DNA sequencing.

### Culture of primary human coronary artery smooth muscle cells

Primary human coronary artery smooth muscle cells (HCASMC, donor 1522) purchased from Cell Applications (San Diego, CA, USA) were used between passages 2 and 5. The cells were cultured in commercial HCASMC growth media or basal media (Cell Applications) in a humidified incubator under an atmosphere of 5% CO_2_ at 37 °C. Basal medium contains essential and non-essential amino acids, vitamins, inorganic salts, organic compounds, and trace elements, but does not contain the growth supplements necessary for cell proliferation such as specific growth factors, antibiotics and serum. For experiments, the cells were harvested with trypsin/EDTA solution (0.025% trypsin, 0.01% EDTA, in PBS), centrifuged at 220 g for 5 min and plated overnight at a density of 1 × 10^5^ cells mL^−1^ in 6-, 12- or 96-well plates using volumes of 2 mL, 1 mL or 50 μL, respectively. For microscopy, cells were plated at a density of 7.5 × 10^4^ cells mL^−1^ in eight-well chamber slides (734 2050, VWR) in a volume of 300 μL, or 96-well plates in 200 μL media. Before treatment, the cell media was removed, and the cells washed with warm (37 °C) Hanks’ buffered salt solution (HBSS).

### Cell adhesion assays

Wt and L37AY40A AN (0.5 μM; 25 μL per well) was added to a 96-well black microplate (sterile, F-bottom, Costar, #3603, Corning, NY, USA) in the presence or absence of FN (2 μg mL^−1^; 25 μL per well) prior to washing with PBS followed by blocking with 1% (w/v) denatured BSA in PBS for 1 h and incubation overnight at 21 °C. Alternatively, 50 μL wt or L37AY40A AN (0.25 μM) was added to wells pre-coated with FN (1 μg mL^−1^; 50 μL per well) and blocked with 1% (w/v) denatured BSA in PBS, followed by incubation overnight at 21 °C. HCAMSC were added (0.5 × 10^4^ cells per well) and the plates incubated for 1 h at 37 °C. After washing twice with HBSS, the plates were incubated with 50 μM calcein-AM (50 μL per well) for 30 min at 37 °C. Adhesion of dye-loaded cells to AN was determined by measuring fluorescence intensity with λ_ex_ 490 nm and λ_em_ 520 nm, using a SpectraMax i3x microplate reader (Molecular Devices, San Jose, CA).

### ELISA

#### Detection of AN in isolated ECM

HCASMC (1.5 × 10^4^ cells, 96 well plate) were cultured in growth media for 1 week to establish native ECM. The plates were then rinsed twice with PBS followed by incubation with 1% sodium deoxycholate at 21 °C for 2 × 20 min to remove cells. After washing twice with PBS, the native ECM remaining on the plates was then blocked with 1% (w/v) BSA in PBS and treated with 0–20 μM wt and L37AY40A AN at 4 °C overnight. Residual materials were then removed from the plates by washing with PBS followed by blocking with 1% (w/v) BSA in PBS. Plates were then incubated with anti-His tag mAb 18,184 (1:500 dilution) antibody at 4 °C overnight. After that, plates were rinsed twice with PBS and incubated with anti-mouse IgG secondary antibody at 21 °C for 1 h. After washing twice with PBS, the plates were then incubated with ABTS (2,2'-azinobis-3-ethylbenzothiazoline-6-sulfonic acid; 2 mM) and H_2_O_2_ (30% w/v), mixed in 1000:1 ratio. Optical absorbance of the samples was measured at 405 nm using a microplate reader.

#### Detection of isolated plasma FN mixed with AN

Wt and L37AY40A AN (0–20 μM) were mixed with plasma FN (1 μg mL^−1^) in a 96-well cell culture plate followed by incubation at 21 °C overnight. Alternatively, wt and L37AY40A AN (0–20 μM) was added to wells pre-coated with FN (50 μL, 20 μg mL^−1^) followed by incubation at 21 °C overnight. In both cases wells were subsequently blocked with 1% (w/v) BSA in PBS for 1 h followed by incubation with primary antibodies (pAb 2413, mAb A32, or mAb 18,184; 1:500 dilutions) at 4 °C overnight. Plates were rinsed twice with PBS and incubated with IgG secondary antibodies at 21 °C for 1 h. After washing twice with PBS, the plates were then incubated with ABTS/H_2_O_2_ (as above), and the absorbance of the samples measured at 405 nm using a microplate reader.

### Immunofluorescence microscopy

#### Detection of FN in HCASMC incubated with wt or L37AY40A AN

HCASMC (2.25 × 10^4^ cells) were cultured in eight-well chamber slides overnight and then incubated with growth media containing 200 μL wt or L37AY40A AN (30 μM) for 48 h. Alternatively, cells were incubated with basal media containing 200 μL wt or L37AY40A AN (30 μM) for 24 h. In both cases, cells on slides were washed, fixed with 4% (v/v) formaldehyde at 37 °C for 15 min, permeabilized with 0.5% (v/v) Triton X-100 in PBS on ice for 5 min and blocked with 1% (v/v) BSA in PBS for 1 h. Primary antibodies (pAb 2413 or mAb 3E2) were added in 1:500 dilutions followed by incubation at 4 °C overnight. The slides were then rinsed three times with PBS and incubated with either anti-mouse or anti-rabbit IgG conjugated with Alexa Fluor 488 antibody or Alexa Fluor 594 antibody (1:500 dilution) in 1% (v/v) BSA in PBS at 21 °C for 1 h followed by three rinses with PBS and counterstaining with 1 μg mL^−1^ DAPI in PBS in the dark at 21 °C for 10 min. Cover slips were added after three washes with PBS, and samples imaged using a fluorescence microscope (Olympus, Japan) equipped with cellSense Entry v1.5 software. Exposure times were 100 ms for the FN 2413, 3E2, and His-tag antibodies and 10 ms for DAPI.

#### Detection of AN and FN in isolated ECM

HCASMC (2.25 × 10^4^ cells) were cultured in eight-well chamber slide for 1 week to establish native ECM. The plates were then rinsed twice with PBS followed by incubation with two aliquots of 1% sodium deoxycholate at 21 °C for 20 min to induce cell lysis, each followed by two washes with PBS. The slides were then blocked with 1% (v/v) BSA in PBS at 21 °C for 1 h, washed twice with PBS, and then incubated with 0.1 μM wt or L37AY40A AN, followed by incubation with primary antibodies (pAb 2413 or mAb 18,184) at 4 °C overnight in 1:500 dilutions. The slides were rinsed twice with PBS then incubated with either anti-mouse or anti-rabbit IgG conjugated with Alexa Fluor 488 antibody or Alexa Fluor 594 antibody (1:500 dilution) at 21 °C for 1 h followed by three rinses with PBS. Cover slips were then added, and the samples imaged using a fluorescence microscope as above.

### Immunoblot analysis

HCASMC (2 × 10^5^ cells) were cultured in 6-well plates overnight (2 mL growth media per well) and then incubated in growth media containing 15–30 μM wt or L37AY40A AN for 48 h (1.5 mL per well). The plates were then rinsed with PBS followed by incubation with 150 μL RIPA Lysis Buffer (25 mM Tris HCl, pH 7.6; 150 mM NaCl; 1% NP-40; 1% sodium deoxycholate; 0.1% SDS) including protease inhibitor cocktail (P8340) in 1:100 dilution for 5 min. Samples were then transfered to 1.5 mL tubes, centrifuged at 14,000*g* for 10 min at 4 °C, and the protein supernatant collected. For detection of IL-6, supernatants from cells cultures in basal media containing 200 μL wt or L37AY40A AN (15 or 30 μM) for 24 h were harvested by centrifugation. Samples were analyzed (10 μg protein per lane) by SDS–PAGE using 3–8% NuPAGE Tris acetate gels according to the manufacturer's instructions. After separation, the proteins were transferred to PVDF membranes using an iBlot system. Membranes were then blocked with 1% (w/v) BSA in TBST and probed with primary antibodies: pAb 2413 (FN, 1:1000), mAb 8929 (β-actin, 1:1000), pAb 6672 (IL-6, 1:5000). The membranes were then washed twice with TBST, and incubated for 1 h with anti-mouse or anti-rabbit IgG secondary antibodies at 21 °C. Unbound secondary antibodies were removed by washing, before detection of immune complexes using Western Lightning Plus ECL reagent, and a Sapphire Biomolecular Imager (Azure Biosystems) to acquire images.

### Proteomics analysis of conditioned media

Supernatants (300 μL) containing conditioned media from cells exposed ± 30 μM AN (wt or L37AY40A AN) for 24 h in basal media as described above were loaded on 1 mL HisTrap columns (Cytiva life science) equilibrated in 25 mM HEPES–NaOH pH 7, 500 mM NaCl, 0.25 mM EDTA, 0.25 mM EGTA, 20% v/v glycerol, 5 mM β mercaptoethanol to remove AN. The flow-through was concentrated to 300 μL and mixed with 200 μL 8 M urea (in 0.1 M Tris, pH 8.5) in a spin filter (10,000 kD MWCO, Vivacon 500, Sartorius), then centrifuged at 14,000*g* for 25 min at 20 °C. 400 μL 8 M urea in Tris buffer containing 50 mM DTT was added and incubated at 20 °C for 30 min. The filter was again centrifuged (14,000*g* for 25 min at 20 °C), before addition of 400 μL 8 M urea in Tris buffer containing 50 mM iodoacetamide, and incubated for another 30 min at 20 °C in the dark. Samples were centrifuged as above and 400 μL 1.6 M urea in 0.1 M Tris (pH 8.0) added. After repeating this centrifugation step, 100 μL 1.6 M urea in 0.1 M Tris (pH 8.0) and 1 μL trypsin (0.1 μg μL^−1^) were added followed by incubation overnight at 20 °C. Released peptides were pooled from the flow-through after centrifugation at 14,000*g* for 10 min, and the flow-through from a subsequent wash of the spin filter with 50 μL 0.5 M NaCl. Samples were subjected to stage-tip solid-phase extraction on C18 discs as described previously and analyzed on a Bruker TIMS-TOF PRO mass spectrometer (Bruker Daltonics) in the positive ion mode with a Captivespray ion source on-line connected to a Dionex Ultimate 3000RSnano chromatography systems (Thermo Fisher Scientific). Peptides were separated on a 25 cm × 75 µm Aurora column (Ion optics) at 60 °C with a solvent gradient over 42 min, using acetonitrile with 0.1% formic acid as eluent at a flow rate of 600 nL min^−1^. The mass spectrometer was operated in DIA PASEF mode with 0.53 s cycle time and TIMS ramp time of 100 ms. MS scan range was set to 100–1700 m*/z*. Database searches were performed using DIA-NN^52^ version 1.8 with a spectral library generated in silico from the human UniProt reference proteome (UP000000558) using the following parameters: trypsin with 1 missed cleavage, methionine oxidation and N-terminal acetylation (variable modifications), cysteine carbamidomethylation (fixed modification), MS1 accuracy ± 10 ppm, precursor false discovery rate (FDR) 1%. Post-processing and statistical analysis on triplicate samples of culture supernatants was performed using Perseus (version 1.6.15.0), with a permutation based t-test (FDR 0.05, S0 0.75) performed to determine up- and- downregulated proteins in supernatants from cell cultures treated with AN (wt or L37AY40A) relative to control cells (Supplementary data S1). Up/down regulated proteins included in the matrisome database^53^ are listed in Tables 1 and 2.

### Metabolic activity

HCASMC (0.5 × 10^4^ cells) were cultured in 96-well plates overnight and then incubated in 100 μL growth media containing 15–30 μM wt or L37AY40A AN for 48 h. Cells were then washed with HBSS and re-incubated with 100 μL growth media containing 10 μL MTS regent ((3-(4,5-dimethylthiazol-2-yl)-5-(3-carboxymethoxyphenyl)-2-(4-sulfophenyl)-2H-tetrazolium)) for 4 h at 37 °C. The absorbance at 490 nm was then measured with a microplate reader.

### Lactate dehydrogenase (LDH) release

HCASMC (1 × 10^5^ cells) were cultured in 12-well plates overnight (1 mL growth media per well) and then incubated in 1 mL growth media containing 15–30 μM wt or L37AY40A AN for 48 h. After incubation, the media was collected and the cells were washed with HBSS and lysed with 1 mL npH_2_O. Both media and cell lysate solutions were centrifuged at 448*g* for 5 min at 4 °C to remove cell debris. Ten μL of supernatant were then mixed with 200 μL of reaction reagent containing 0.15 mg mL^−1^ NADH and 2.5 mM sodium pyruvate in PBS. LDH activity was measured by detecting the decrease in absorbance at 340 nm for 30 min at 5 min intervals with a microplate reader. Cell viability was calculated as intracellular LDH activity compared to the total intra- and extracellular LDH activity, expressed as a percentage.

### Cell proliferation assay

HCASMC (1 × 10^5^ cells) were plated into 12-well plates overnight and then incubated in 1 mL growth media containing 15–30 μM wt or L37AY40A AN for 48 h at 37 °C. Cells were then released from the plate using trypsin–EDTA, prior to cell counting using a hemocytometer with trypan blue staining to delineate non-viable cells. Total cell number was then calculated for each sample in triplicate.

### qPCR analysis

HCASMC (1 × 10^5^ cells in 1 mL/well) cultured overnight in a 12-well plate, and then incubated in growth media containing 30 μM wt or L37AY40A AN for 2 h. Total RNA was extracted with the RNeasy kit (Qiagen), and RNase free DNase (Qiagen) was used for DNase digestion. 600 ng RNA was used for cDNA synthesis using Quantinova Reverse Transcription Kit (Qiagen). For qPCR analysis, 20 μL reaction mixtures (containing 10 μL SYBR® GreenER™, 2 μL primer (10 pmol μL^−1^), 0.4 μL ROX reference dye and 4 μL cDNA) were incubated at 50 °C for 2 min followed by 95 °C for 10 min (hold stage); 95 °C for 15 s followed by 60 °C for 1 min (PCR stage, repeated 40 times); 95 °C for 15 s followed by gradual increase from 60 to 95 °C by 0.075 °C s^−1^ (melt curve stage). The reaction was performed on the QuantStudio™ 5 real-time PCR system (Applied Bio-systems). Primers used were (5ʹ–3ʹ): FN1 (F: GGTGACACTTATGAGCGTCCTAAA; R: CCCATCAGCAGGAACACCTT), PCNA (F: AGGCACTCAAGGACCTCATCA; R: GAGTCCATGCTCTGCAGGTTT), CCNB1 (F: AGCTGCTGCCTGGTGAAGAG; R: GCCATGTTGATCTTCGCCTTA), CCNA1 (F: GCACCCTGCTCGTCACTTG; R: CAGCCCCCAATAAAAGATCCA). Relative mRNA concentrations of the genes of interest were normalized to the expression of genes encoding 18S ribosomal RNA (18S rRNA) (F: GAGGATGAGGTGGAACGTGT; R: TCTTCAGTCGCTCCAGGTCT) and beta-2-microglobulin (B2M) (F: AGATGAGTATGCCTGCCGTG; R: GCGGCATCTTCAAACCTCCA) housekeeping genes. Experiments were performed in triplicates and data analysis was carried out using the 2^−ΔΔCT^ method.

### Heparin binding assay

Experiments were performed in three different ways: i) Plasma FN (20 μg mL^−1^) or wt or L37AY40A AN (0.25 μM and 1 μM) in 50 μL was added to a 96-well black microplate (sterile, F-bottom, Costar, 3603, Corning, NY, USA), which was incubated overnight, followed by blocking with 1% (w/v) BSA in PBS. ii) AN (wt or L37AY40A, 50 μL, 1 μM) was added to wells pre-coated with FN (20 μg mL^−1^) as outlined above, and then incubated at 21 °C overnight. iii) 50 μL plasma FN (1 μg mL^−1^) was co-incubated with 0.25 μM AN (wt or L37AY40A) in a 96-well black microplate overnight followed by blocking with 1% (w/v) BSA in PBS, pH 7.4. In all experiments plates were subsequently incubated with 50 μL fluorescein-labeled heparin (0.004 mg mL^−1^) overnight at 21 °C in the dark and analyzed using a microplate reader with λ_ex_ 495 nm and λ_em_ 525 nm (9 nm slit width), with three spectra averaged.

### Turbidity and sedimentation assays

Wt AN (2.5–40 μM) or L37AY40A AN (40 μM) was mixed with FN (0.4 mg mL^−1^; 1.4 μM) before measuring the turbidity of the solution at 550 nm at 1 min intervals for a period of 40 min at 21 °C, using a Spectra Max i3x microplate reader (Molecular Devices, San Jose, CA, USA). In the sedimentation assay, wt or L37AY40A AN (40 μM) and FN (1 μM) were mixed in a final volume of 20 μL in sodium phosphate buffer (10 mM, pH 7.4) containing 5 mM EDTA and incubated overnight at 21 °C. After incubation, the samples were centrifuged at 20,000 g for 10 min at 4 °C. The insoluble pellets were then washed with 20 μL sodium phosphate buffer (10 mM, pH 7.4). All samples were analyzed by SDS-PAGE under reducing conditions.

### CD spectroscopy

CD spectra were acquired on a Jasco-815 CD Spectrometer (Jasco Corporation, Japan), as previously^51^. Far-UV CD spectra of 0.1 mg mL^−1^ wt and L37AY40A AN was recorded between 190–260 nm, in a 1 mm path length cell at 25 °C, at 1 nm intervals with a 1 nm bandwidth, and a scan speed of 50 nm min^−1^, with six scans averaged for each spectrum.

### SAXS analysis

SAXS measurements were performed as previously^51^ on an optimized NanoSTAR SAXS instrument^54^, with wt and L37AY40A AN (100 μM; 0.93 mg mL^−1^) in 10 mM phosphate buffer (pH 7.4). The SAXS data is displayed as a function of the scattering vector, *q*. A scaling factor was used to compare data between wt and L37AY40A AN. The radius of gyration, *R*g, was obtained by performing Guinier fits.

### Heparin-affinity chromatography

Wt and L37AY40A AN (80 μM) in 100 μL 20 mM sodium phosphate buffer, pH 7.4) was loaded onto a Hitrap heparin high-performance column (GE healthcare, Boston, MA) equilibrated in 20 mM sodium phosphate buffer (pH 7.4) on an Äkta Prime chromatography system (GE Healthcare). The proteins were eluted with a gradient of 0–500 mM NaCl in 20 mM sodium phosphate buffer (pH 7.4) at a flow rate of 1 mL min^−1^. Protein elution was monitored by UV detection at 280 nm.

### Errors and statistical analyses

Statistical analyses were performed using Graphpad Prism (version 9; GraphPad Software, San Diego, USA) by 2-way ANOVA with Tukey's multiple comparison test. Quantitative data are presented as mean ± SD from at least three independent experiments, with p < 0.05 (indicated by * or #) taken as being statistically significant, and ‘ns’ indicating an absence of statistical significance. Analysis of pixel intensities in microscopy images was carried out using R (version 4.1.3). For each condition, pixel intensities from three images (each derived from an independent experiment) were pooled. Each pixel was loaded into a numeric r vector using the readTIFF function from the tiff package. The median of all pixels was then calculated via the summary function, and statistical analysis was performed using one-way ANOVA with post-hoc Tukey's multiple comparison tests. The boxplots were calculated from a random subset of the pixel data (300,000 per sample) and the ggplot2 library.

## Supplementary Information

Supplementary Information 1.

Supplementary Information 2.

Supplementary Information 3.

## Data Availability

This article contains Supplementary information. Raw mass spectrometry data and associated database search engine output files have been deposited to the ProteomeXchange Consortium via the PRIDE partner repository with the dataset identifier PXD033732.

## Abbreviations

ABTS: 2,2'-Azino-bis(3-ethylbenzothiazoline-6-sulfonic acid)
AN: Anastellin
CD: Circular dichroism
DAPI: 4′,6-Diamidino-2-phenylindole
ECM: Extracellular matrix
FN: Fibronectin
HCASMC: Human coronary artery smooth muscle cells
IL-6: Interleukin 6
L37AY40A AN: An isoform of anastellin with both Leu37 and Tyr40 mutated to Ala residues
LDH: Lactate dehydrogenase
mAb: Monoclonal antibody
MTS: (3-(4,5-Dimethylthiazol-2-yl)-5-(3-carboxymethoxyphenyl)-2-(4-sulfophenyl)-2H-tetrazolium)
pAb: Polyclonal antibody
qPCR: Quantitative real-time PCR
SAXS: Small-angle X-ray scattering
VEGF: Vascular endothelial growth factor
wt: Wild type
