# Supplementary material for: Anastellin impacts on the processing of extracellular matrix fibronectin and stimulates release of cytokines from coronary artery smooth muscle cells
Source: Sci Rep. 2022 Dec 21;12:22051. doi: 10.1038/s41598-022-26359-9 (PMC9772232; doi:10.1038/s41598-022-26359-9)

Supplementary information

Anastellin impacts on the processing of extracellular matrix fibronectin and stimulates release of cytokines from coronary artery smooth muscle cells

Jianfei He[1], Jonas Hyld Steffen[1], Peter Waaben Thulstrup[2], Jannik Nedergaard Pedersen[3,#], Max B. Sauerland[1], Daniel E. Otzen[3], Clare L. Hawkins[1], Pontus Gourdon[1], Michael J. Davies[1*], Per Hägglund[1*]

[1]Department of Biomedical Sciences, University of Copenhagen, Copenhagen, Denmark

[2]Department of Chemistry, University of Copenhagen, Copenhagen, Denmark

[3]Interdisciplinary Nanoscience Center (iNANO); Department of Molecular Biology and Genetics, Aarhus University, Aarhus, Denmark

[#]Present address: Arla Foods Ingredients Group P/S, Sønderupvej 26, 6920 Videbæk, Denmark

*Joint senior and corresponding authors: M.J. Davies (davies@sund.ku.dk), P. Hägglund (pmh@sund.ku.dk)

List of supporting information:

-Supplementary Figure S1-S16

-Supplementary Data S1-S2 (provided as separate excel files)

Figure S1. Half-violin plots of pixel intensities for the microscopy data with the 2413 and 3E2 antibodies presented in Fig. 1. For each condition, pixel intensities from three images (each derived from an independent experiment) are plotted. Statistical analysis was performed on the median pixel intensity via one-way ANOVA with post-hoc Tukey's multiple comparison tests and the results are indicated by the corresponding p-value, ns > 0.05, * < 0.05.

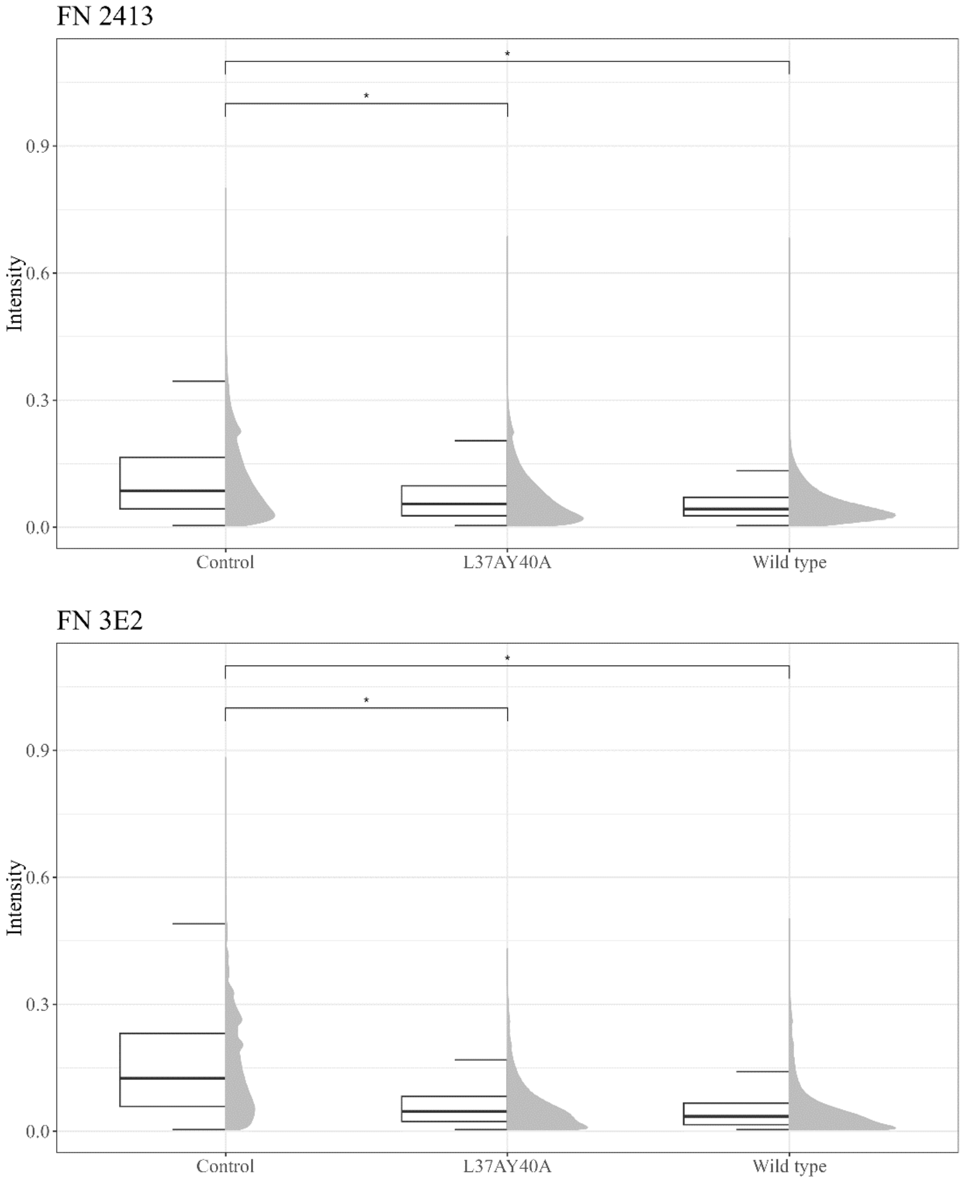

Figure S2. AN influences the structure of isolated matrix laid down by HCASMC cultured in growth media. Primary HCASMCs at an initial density of 2.25×10^4 cells per well in 8-well chamber slides were cultured overnight in growth media and then incubated in growth media containing 30 μM AN (wt or L37AY40A) for 48 h. HCASMCs were then removed with 1% sodium deoxycholate, followed by incubation with primary antibodies at 4 °C overnight. **Panel a:** Total FN was visualized by an anti-FN pAb (2413, 1:500, green channel) and cell-derived FN was detected using a mAb recognizing the EDA module of cell-derived FN (3E2, 1:500, red channel). Overlaid images are shown on the right hand side. Scale bars: 50 μm. **. Panel b:** Half-violin plots of pixel intensities for 3E2 and 2413 staining. For each condition, pixel intensities from three images (each derived from an independent experiment) are plotted. Statistical analysis was performed on the median pixel intensity via one-way ANOVA with post-hoc Tukey's multiple comparison tests and the results are indicated by the corresponding p-value, ns > 0.05, * < 0.05.

a

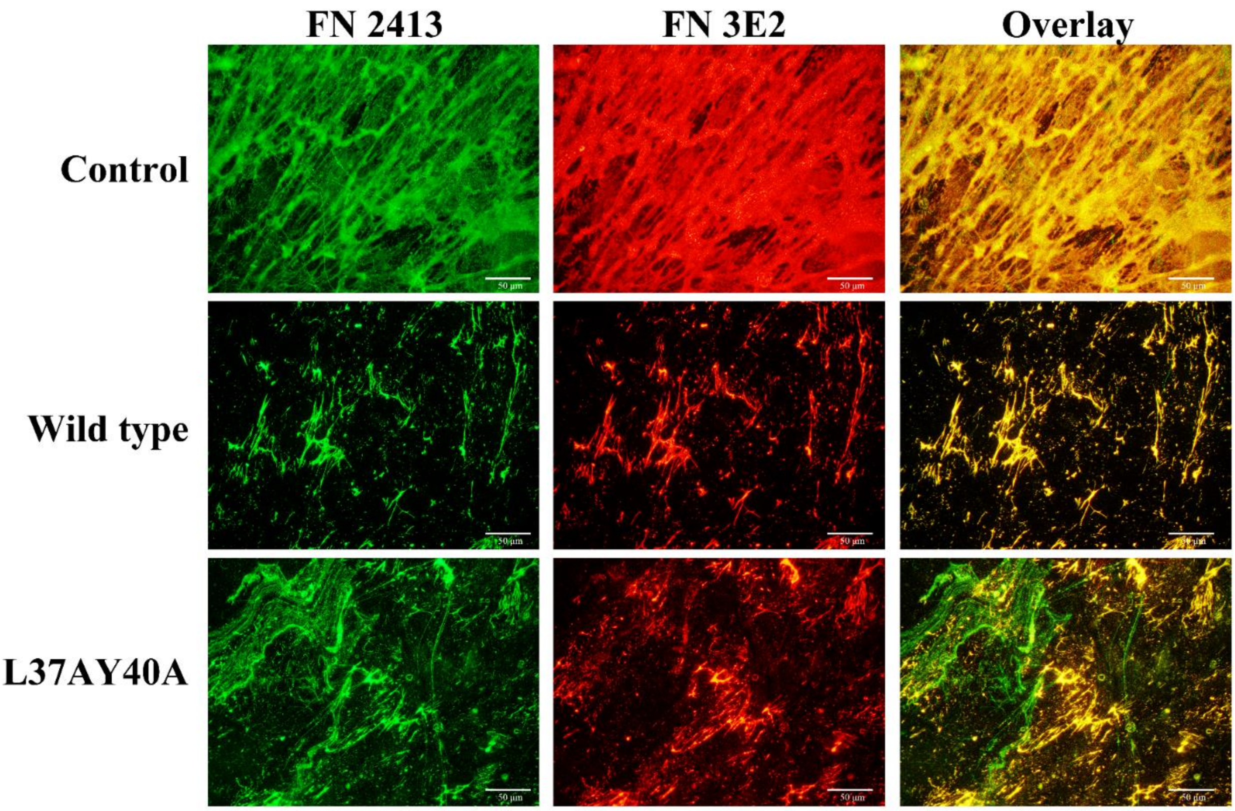

b

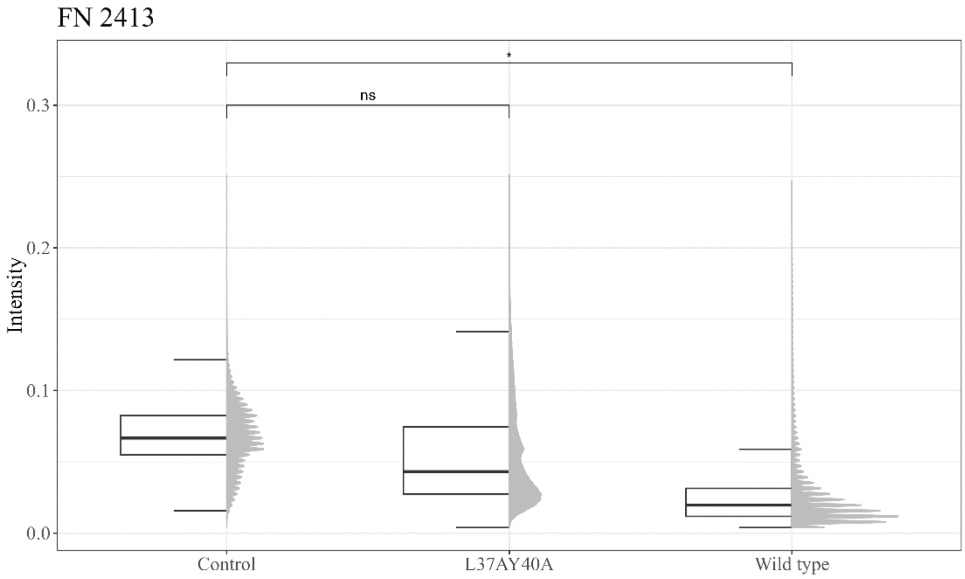
FN 2413
*
ns
Intensity
0.0
0.1
0.2
0.3
Control
L37AY40A
Wild type

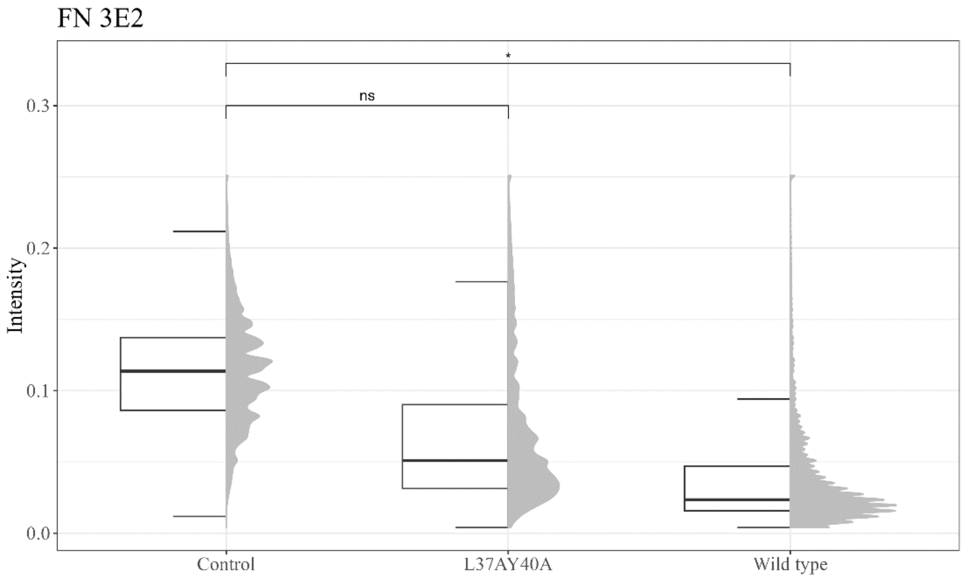
FN 3E2
*
ns
Intensity
0.0
0.1
0.2
0.3
Control
L37AY40A
Wild type

Figure S3. AN binds to FN in the ECM of HCASMC. Primary HCASMCs (initial density 2.25 × 10^4 cells per well) were cultured for 7 days to establish an ECM. The HCASMCs were then removed (1% sodium deoxycholate) and the remaining ECM on the plates incubated with AN (0.01−0.1 μM) overnight. **Panel a**: Total FN visualized using a pAb against FN (2413, 1:500, green channel) and AN using an anti-His tag mAb (1:500, red channel). Overlaid images shown on right. Scale bars: 50 μm. **Panel b**: Quantification of signal from ECM-bound AN by ELISA using mAb His-tag antibody (1:500) as described in the Materials and methods. Data are presented as means ± SD from three independent experiments, expressed as a % of the values detected with the highest concentration (0.1 μM) of wt AN, and analyzed by 2-way ANOVA with Tukey's multiple comparison test * indicates significant difference from the control (no added AN) at the $p < 0.05$ level. # indicates significant differences between wt and L37AY40A AN at the $p < 0.05$ level. 'ns' indicates an absence of significant differences.

Figure S3

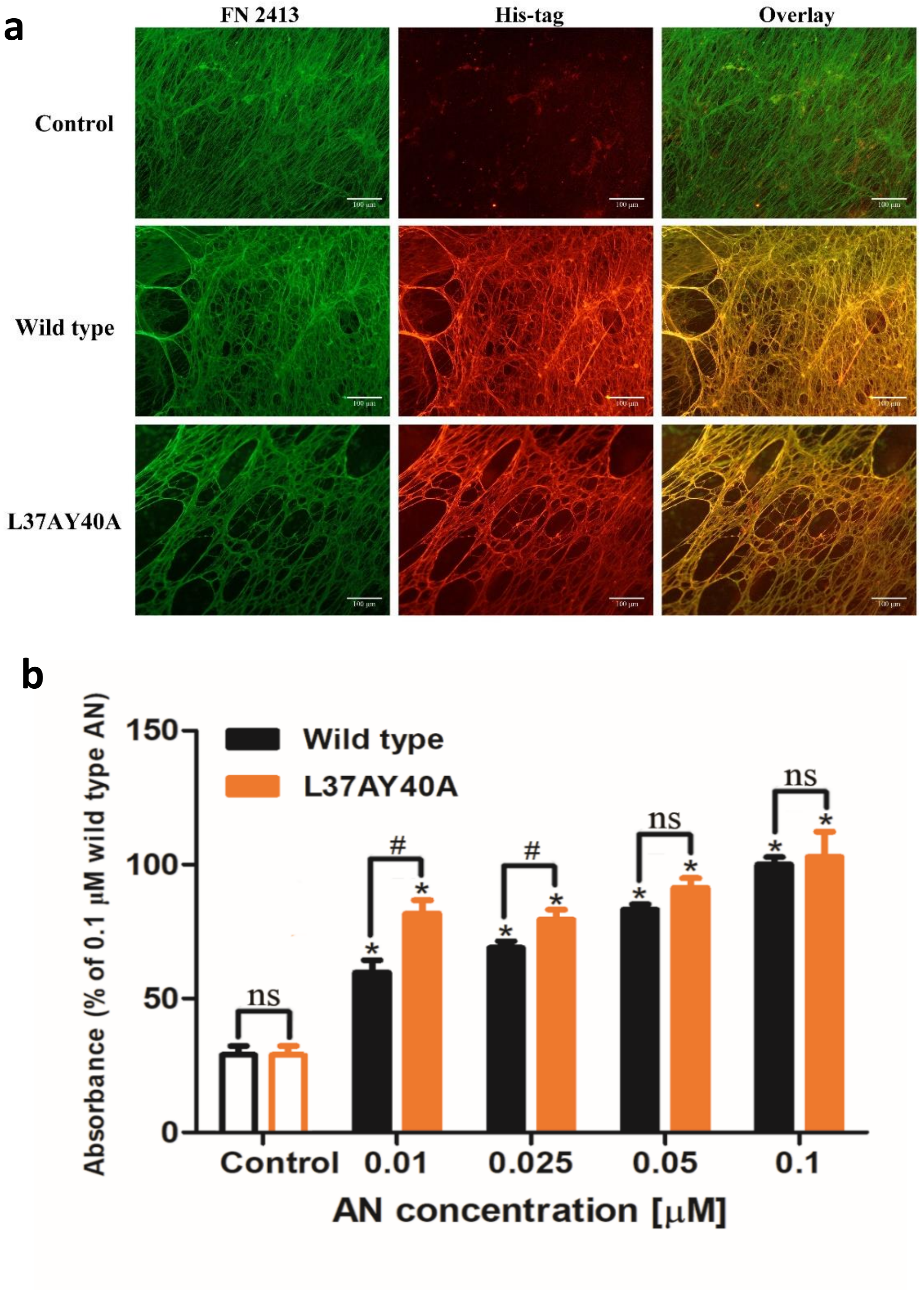
a
FN 2413
His-tag
Overlay
Control
Wild type
L37AY40A
b
Absorbance (% of 0.1 μM wild type AN)
150
100
50
0
Wild type
L37AY40A
ns
#
#
ns
ns
*
Control
0.01
0.025
0.05
0.1
AN concentration [μM]

Figure S4. Half-violin plots of pixel intensities for microscopy data with the 2413 and 3E2 antibodies presented in Fig. 2. For each condition, pixel intensities from three images (each derived from an independent experiment) are plotted. Statistical analysis was performed on the median pixel intensity via one-way ANOVA with post-hoc Tukey's multiple comparison tests and the results are indicated by the corresponding p-value, ns > 0.05, * < 0.05.

a) 0 μg mL^{-1} FN

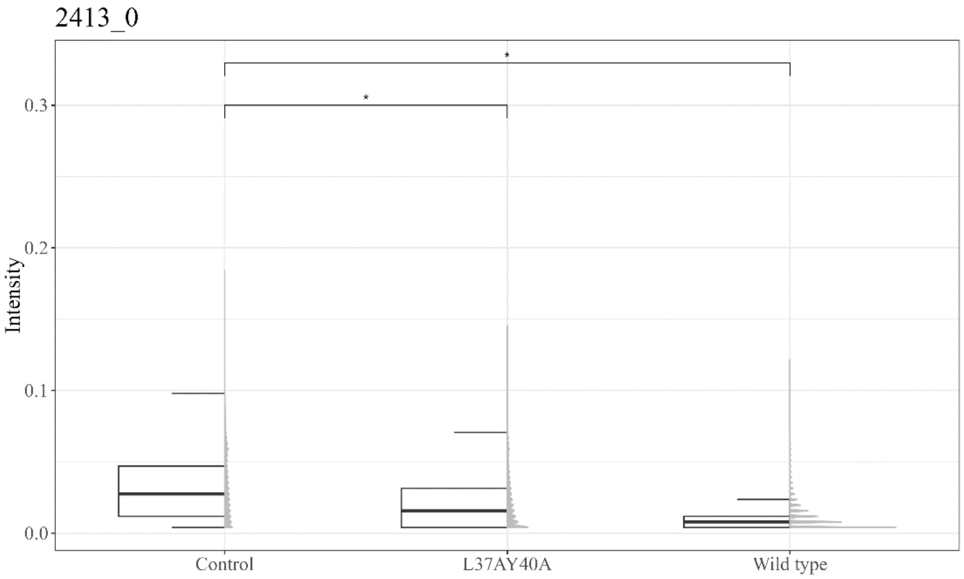

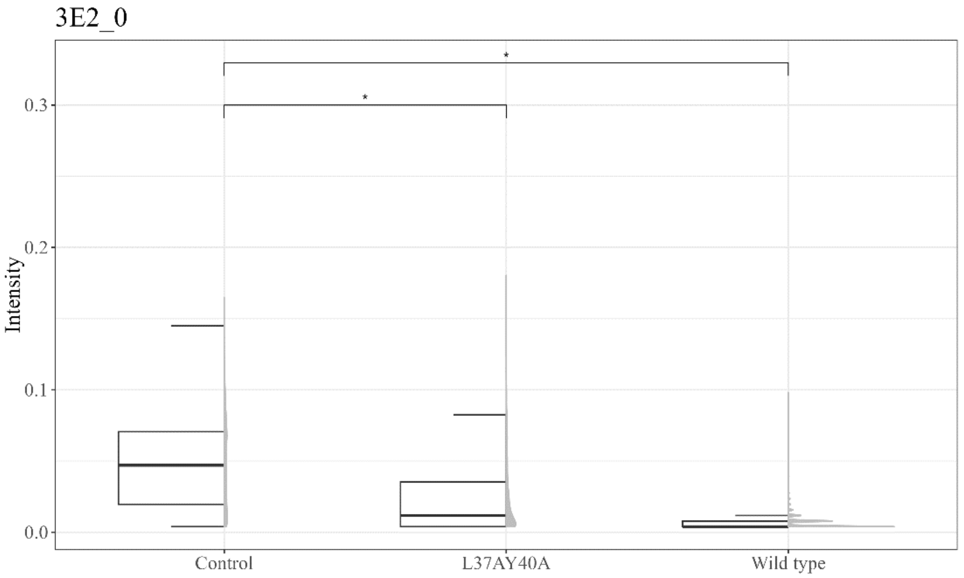

b) 10 μg mL^{-1} FN

2413_10

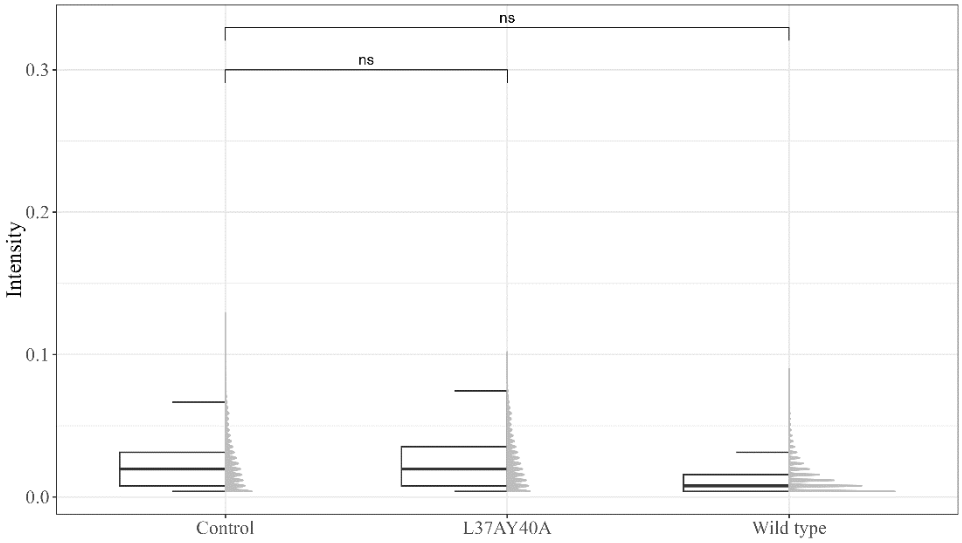

3E2_10

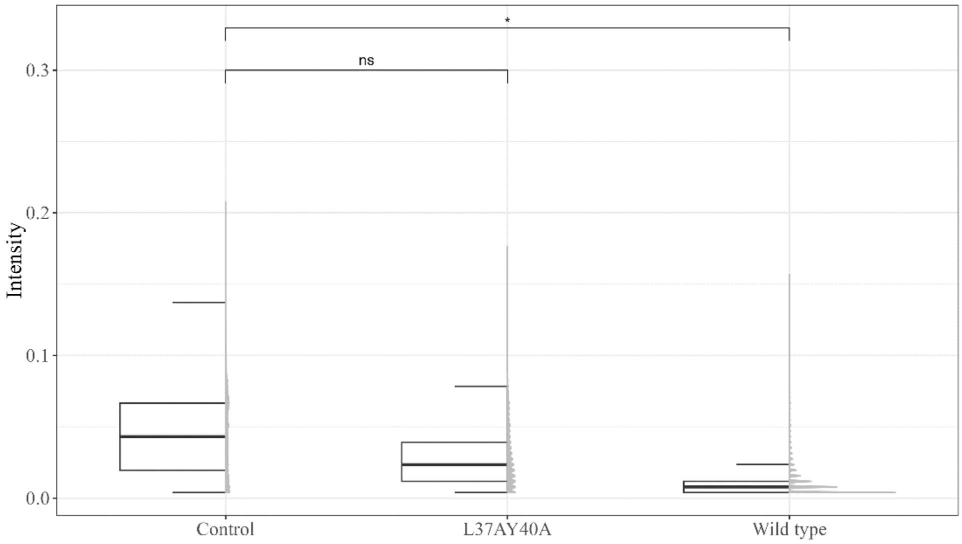

c) 100 μg mL^{-1} FN

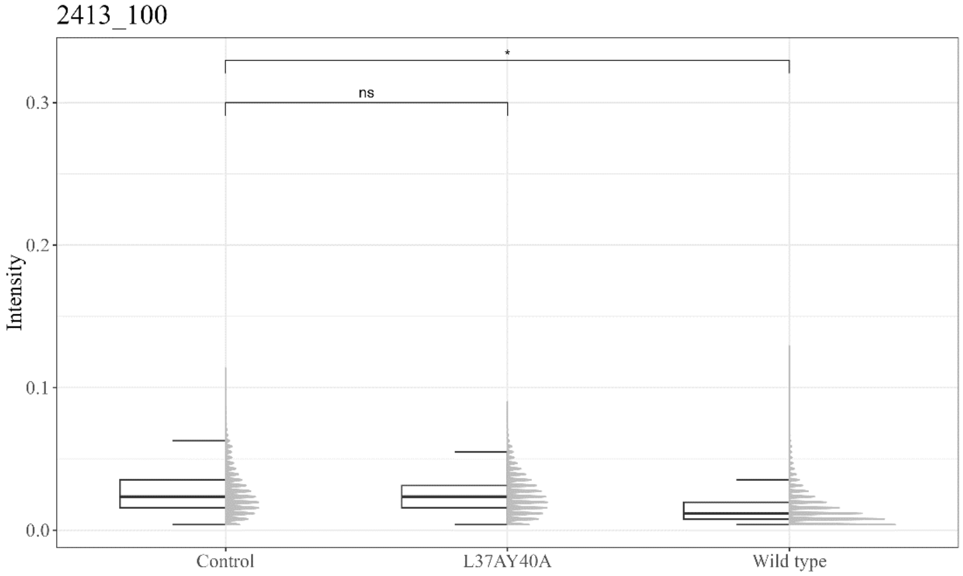

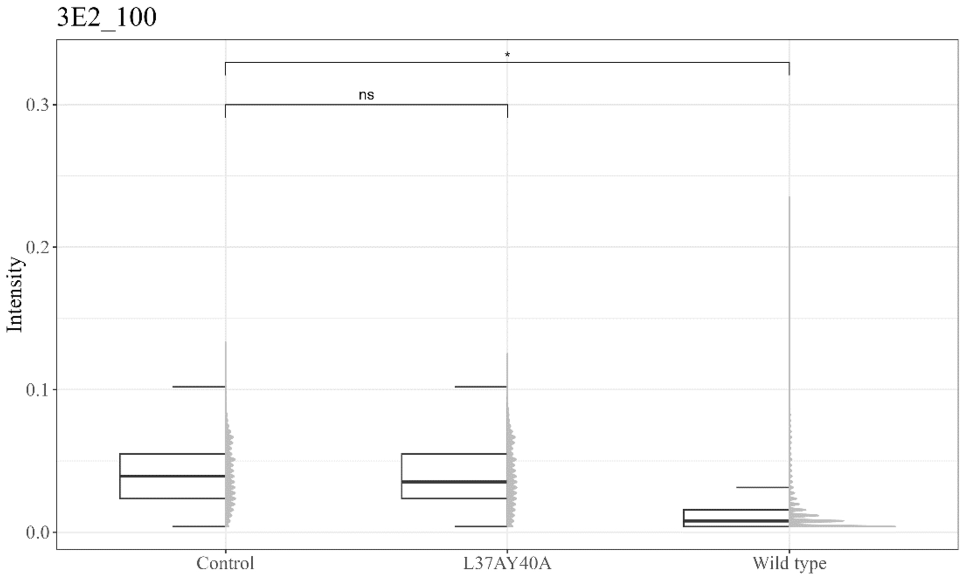

Figure S5. FN detected in cells exposed to AN for 2 h. Primary HCASMC at an initial density of 2.25×10^4 cells per well in 8-well chamber slides were cultured in basal media overnight and then incubated with 30 µM AN (wt or L37AY40A) in basal media for 2 h. HCASMCs on the slides were then fixed and permeabilized followed by incubation with primary antibodies at 4 °C overnight. **Panel a:** Total FN was visualized by anti-FN pAb (2413, 1:500, green channel) and cell-derived FN detected using a mAb that recognizes the EDA epitope (3E2, 1:500, red channel); nuclei were counterstained using DAPI (blue channel). Overlaid images are shown on the right. Scale bars: 20 µm. **Panel b:** Half-violin plots of pixel intensities for the microscopy data with the 2413 and 3E2 antibodies. For each condition, pixel intensities from three images (each derived from an independent experiment) are plotted. Statistical analysis was performed on the median pixel intensity via one-way ANOVA with post-hoc Tukey's multiple comparison tests and the results are indicated by the corresponding p-value, ns > 0.05, * < 0.05.

a)

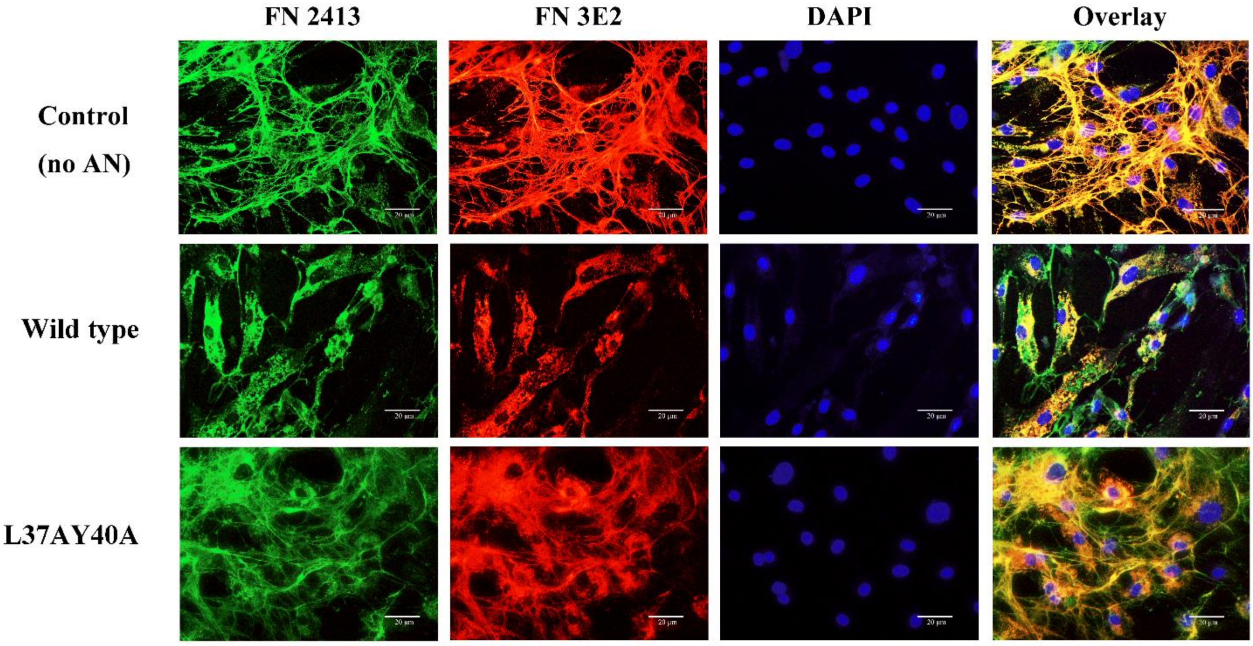

b)

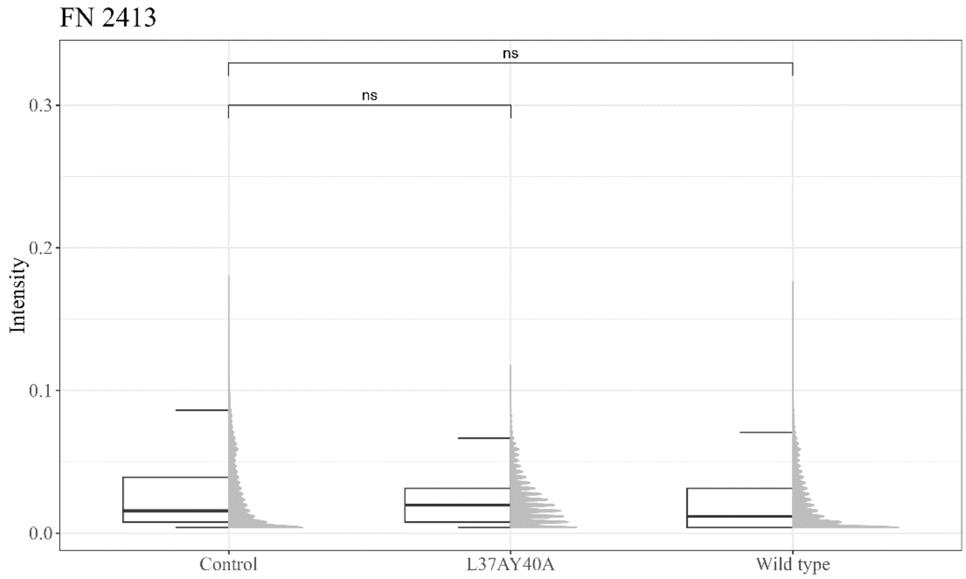
FN 2413
ns
ns
0.3
0.2
0.1
0.0
Intensity
Control
L37AY40A
Wild type

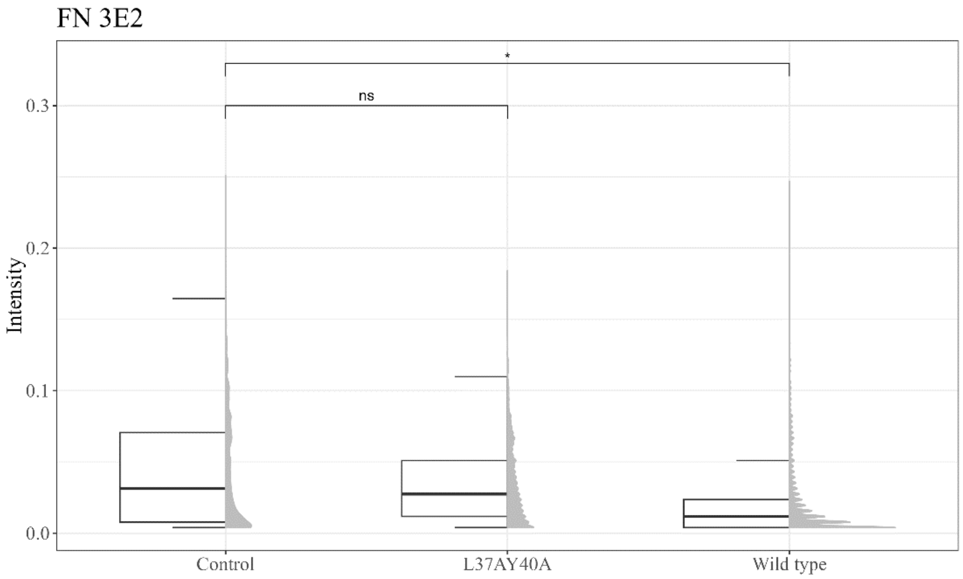
FN 3E2
*
ns
0.3
0.2
0.1
0.0
Intensity
Control
L37AY40A
Wild type

Figure S6. AN has a moderate influence on intracellular levels of FN generated by HCASMC. Cells at an initial density of 2×10^5 cells per well in 6-well plates were cultured overnight, and then incubated in growth media containing either 15 or 30 μM wt or L37AY40A AN for 48 h. Cellular proteins extracted with 1% sodium deoxycholate (10 μg samples of total protein) were loaded on to SDS-PAGE gels under reducing condition, electroblotted onto a PVDF membrane and probed with anti-FN pAb 2413 (**panel a**) or anti-β actin mAb (**panel b**). Lane labeled C represent control samples from cells incubated without AN. **Panel c**: densitometric analysis of the bands from FN following normalization to β-actin, with data expressed as a % compared to the respective non-treated control group with no AN. **Panel d**: mRNA expression profiles of the FN1 gene. HCASMC at an initial density of 10^5 cells per well in 12-well plates were cultured overnight, and then incubated in growth media containing 30 μM wt or L37AY40A AN for 2 h prior to extraction of RNA and qPCR analysis. Data is expressed as a fold change relative to the cells grown without AN after normalization to the housekeeping genes encoding beta-2-microglobulin (B2M) and 18S ribosomal RNA (18S rRNA). Data analysis was carried out using the $2^{-\Delta\Delta CT}$ method. Data in **panel c** and **panel d** are presented as means ± SD from three independent experiments. * indicates significant difference compared to control group by 2-way ANOVA with a Tukey's multiple comparison test, at the $p < 0.05$ level. 'ns' indicates an absence of significant difference between wt and L37AY40A AN. Full images of the membranes are presented in Supplementary Figure S16.

Figure S6

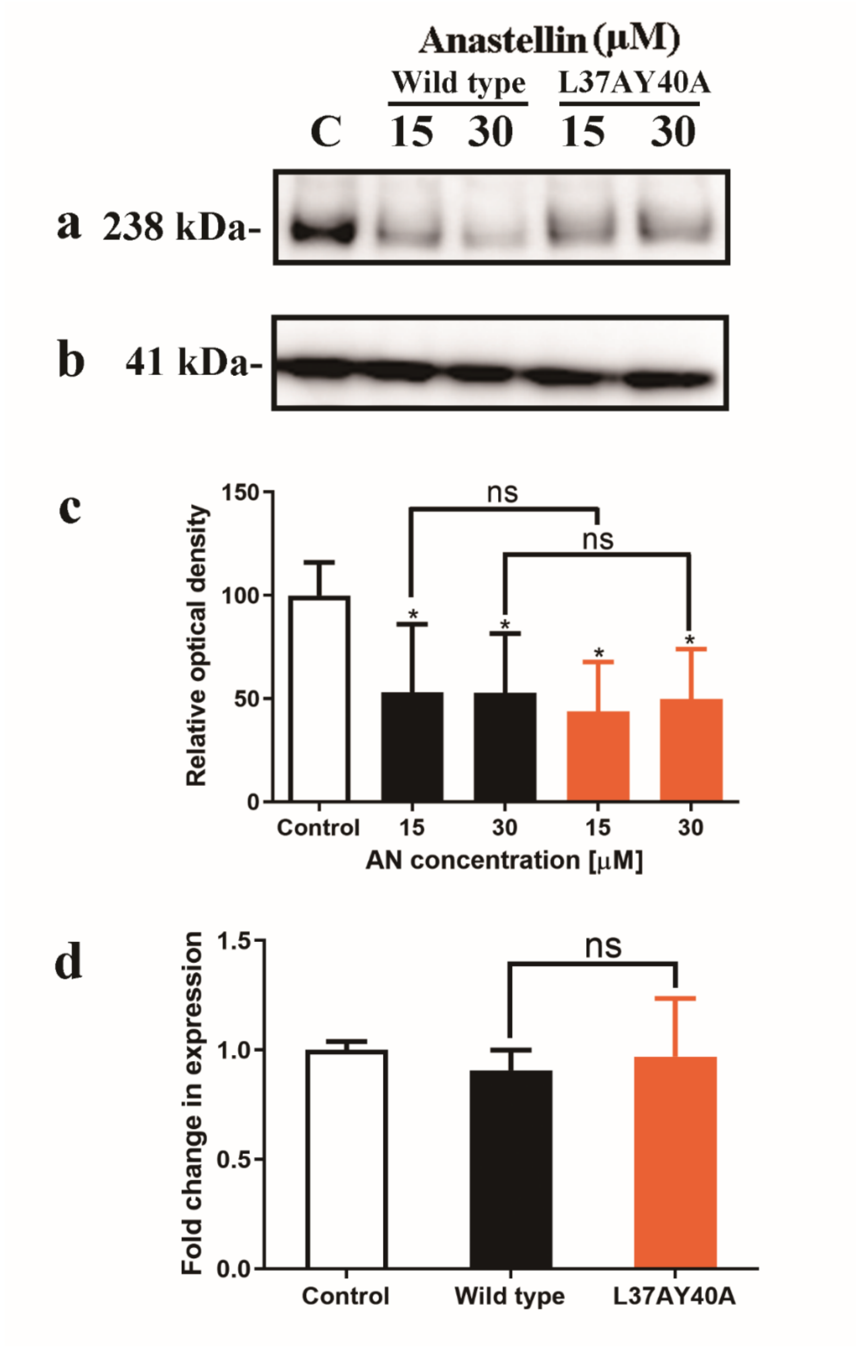

Anastellin (μM)
Wild type
L37AY40A
C
15
30
15
30
a
238 kDa-
b
41 kDa-
c
Relative optical density
150
100
50
0
ns
ns
*
Control
15
30
15
30
AN concentration [μM]
d
Fold change in expression
1.5
1.0
0.5
0.0
ns
Control
Wild type
L37AY40A

Figure S7. AN does not impair viability, metabolic activity and proliferation of HCASMC. **Panal a**: Assessment of cell viability evaluated by LDH release. HCASMC (initial density 1×10^5 cells per well) were plated into 12-well plates for 24 h, followed by incubation in growth media containing wt or L37AY40A AN (15–30 μM) for 48 h at 37 °C. The cell-conditioned media was then removed and collected, and the cells washed and lysed with npH_2O. LDH activity in the conditioned media and cell lysate was assayed as described in the Materials and methods. **Panel b**: Metabolic activity assessed by a MTS assay. Cells cultured in 96-well plates (initial density 0.5×10^4 cells per well) for 24 h, followed by incubation with wt or L37AY40A AN in growth media as described above for 48 h. Cells were subsequently incubated with 100 μL growth media containing 10 μL MTS reagent per well, incubated for 4 h and absorbance detected at 490 nm. **Panel c**: Cell proliferation (initial density 1×10^5 cells per well) was determined after treatment (as above for 48 h) by staining with trypan blue (to exclude dead cells) and cell were counted using a hemocytometer as described in the Materials and methods. Data are presented as means ± SD from three independent experiments and expressed as a % of the control data, then analyzed by 2-way ANOVA with Tukey's multiple comparison test. * indicates significant difference compared to the control at the $p < 0.05$ level. # indicates a significant difference between the two AN forms at the $p < 0.05$ level. 'ns' indicates an absence of significant difference.

Figure S7

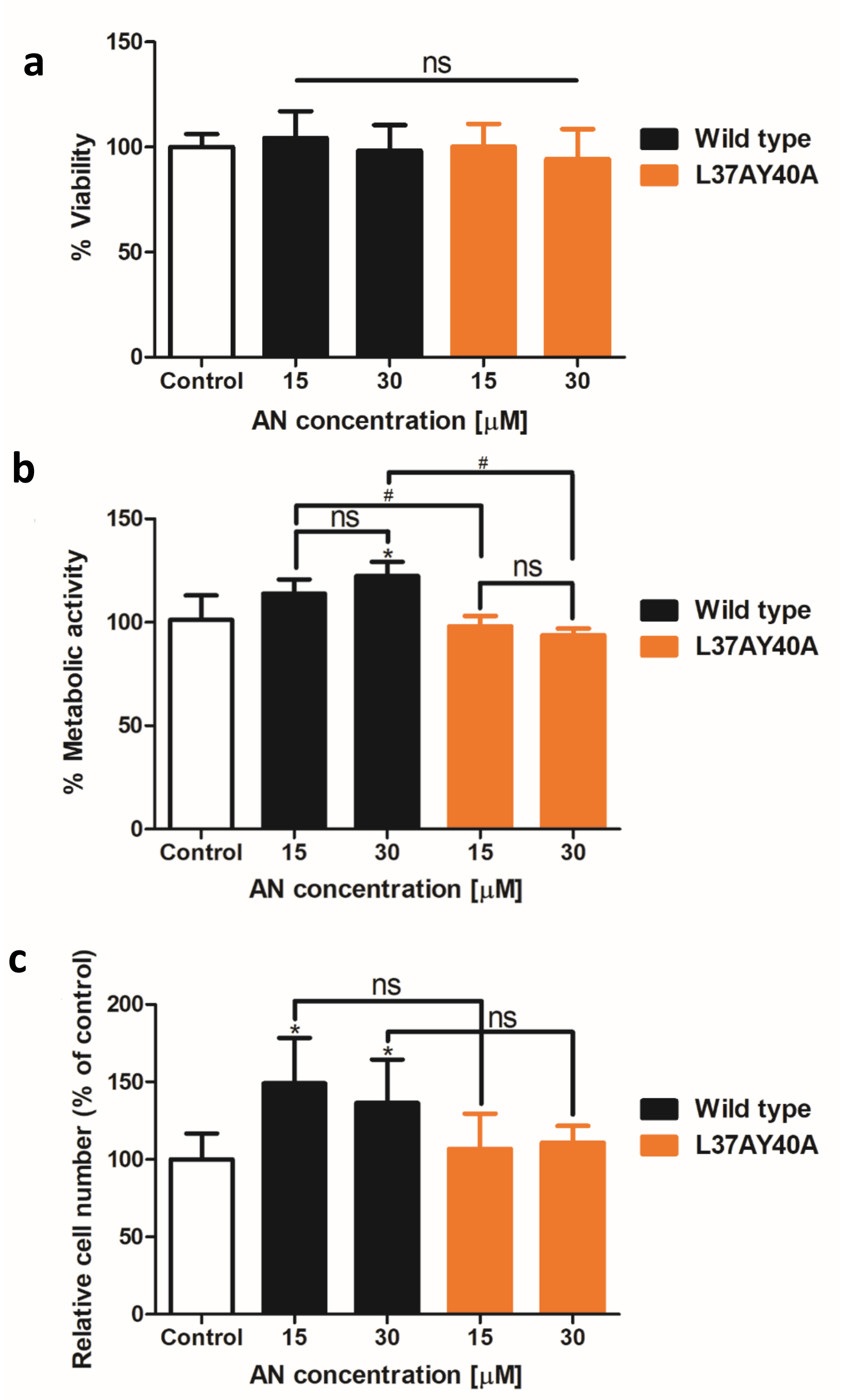
a
150
100
50
0
% Viability
ns
Control
15
30
15
30
AN concentration [μM]
Wild type
L37AY40A
b
150
100
50
0
% Metabolic activity
#
#
ns
*
ns
Control
15
30
15
30
AN concentration [μM]
Wild type
L37AY40A
c
200
150
100
50
0
Relative cell number (% of control)
ns
ns
*
*
Control
15
30
15
30
AN concentration [μM]
Wild type
L37AY40A

Figure S8. Wt and L37AY40A AN do not induce major changes in expression of mitosis-related genes. Cells at an initial density of 10^5 cells per well were cultured in 12-well plates in basal media overnight, and then incubated in growth media containing 30 μM wt or L37AY40A AN for 2 h, followed by isolation of RNA and qPCR analysis of PCNA (**Panel a**), CCNA1 (**Panel b**), and CCNB1 (**Panel c**). Data were expressed as a fold change relative to the cells grown without AN after normalization to the housekeeping genes encoding beta-2-microglobulin (B2M) and 18S ribosomal RNA (18S rRNA). Data analysis was carried out using the $2^{-\Delta\Delta CT}$ method. 'ns' indicates an absence of statistical significance difference between wt and L37AY40A AN by 2-way ANOVA with a Tukey's multiple comparison test.

Figure S8

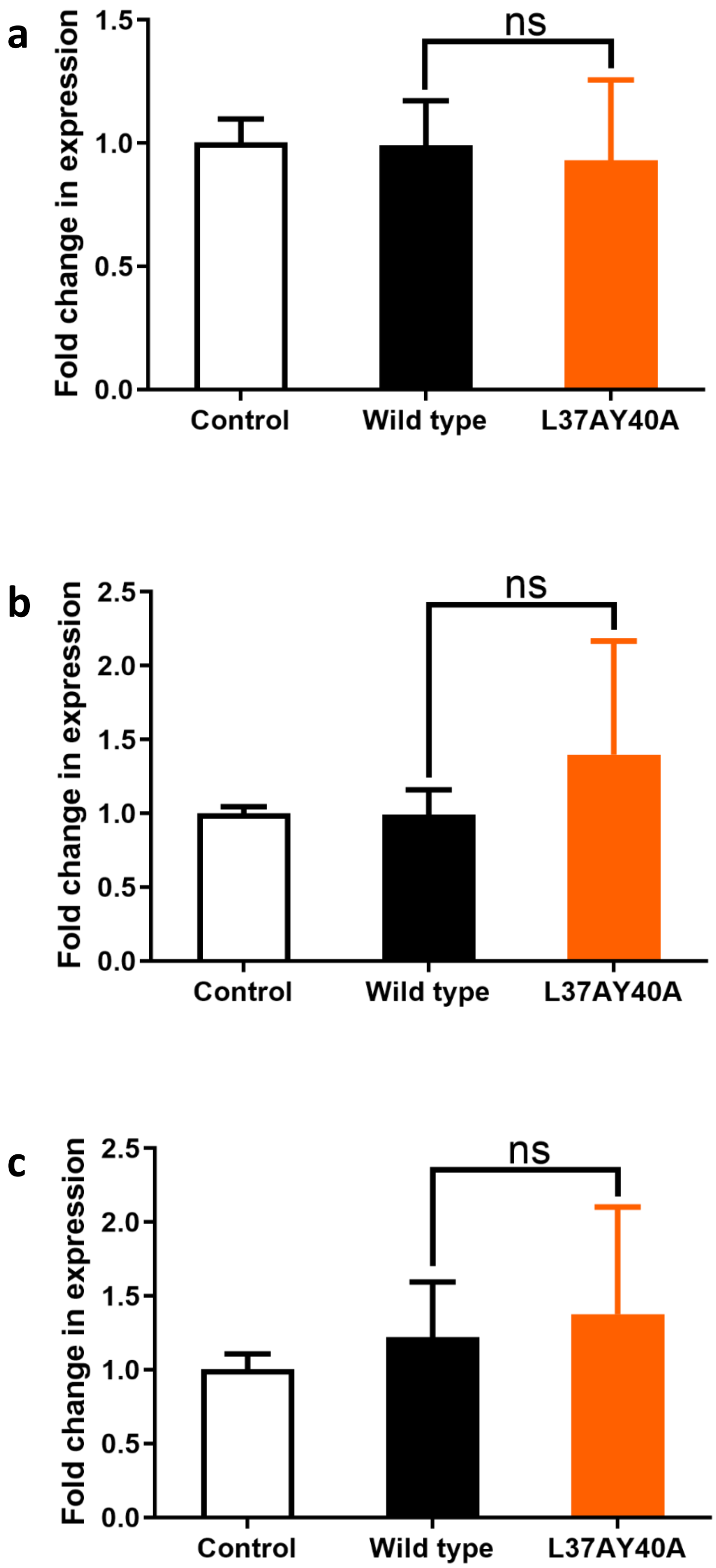
a
b
c
Fold change in expression
ns
Control
Wild type
L37AY40A
0.0
0.5
1.0
1.5
2.0
2.5

Figure S9. Volcano plots displaying up- and downregulated proteins in supernatants from cell cultures exposed to wt AN (a) or L37AY40A AN (b) compared to cells not exposed to AN. Post-processing and statistical analysis with a t-test (FDR 0.05, S0 0.75) on triplicate samples of culture supernatants was performed using Perseus (version 1.6.15.0). X-axis denotes log2-transformed fold change of protein abundance with proteins on the right hand side of the x-axis upregulated in wt (**Panel a**) or L37AY40A AN (**Panel b**) relative to control cells not exposed to AN, and proteins on the left hand side upregulated in control cells relative to wt AN (**Panel a**) or L37AY40A (**Panel b**). The y-axis denotes –log transformed P-values. Uniprot accession numbers are displayed for proteins with log2-transformed fold change >2.

Figure S9

a

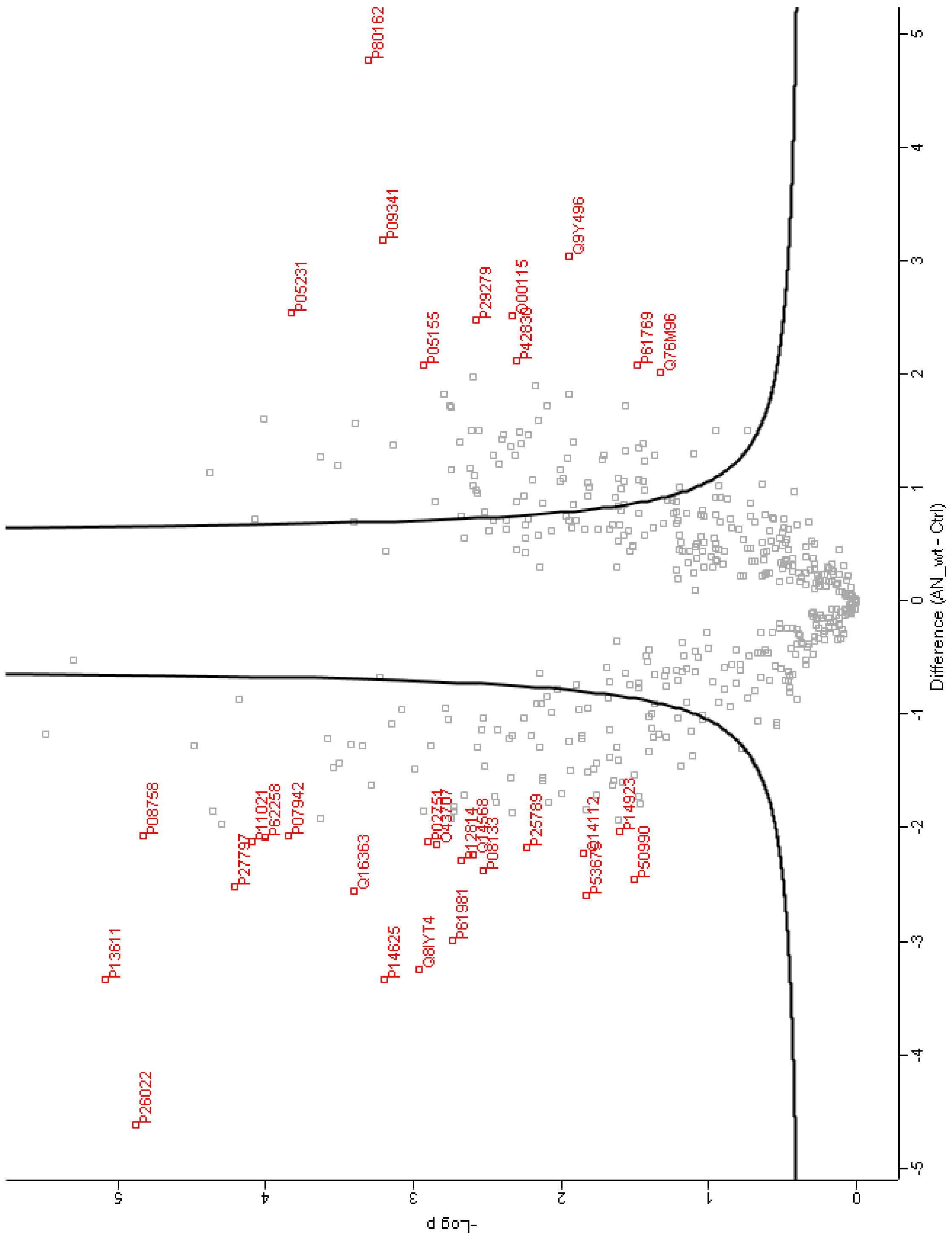

-Log p
Difference (AN_wt - Ctrl)
P80162
P09341
Q9Y496
P05231
P29279
P05155
P61769
Q76M96
P08758
P62258
P07942
P27797
Q16363
P13611
P26022
P14625
Q8IYT4
P61981
P25789
P50990

Figure S9

b

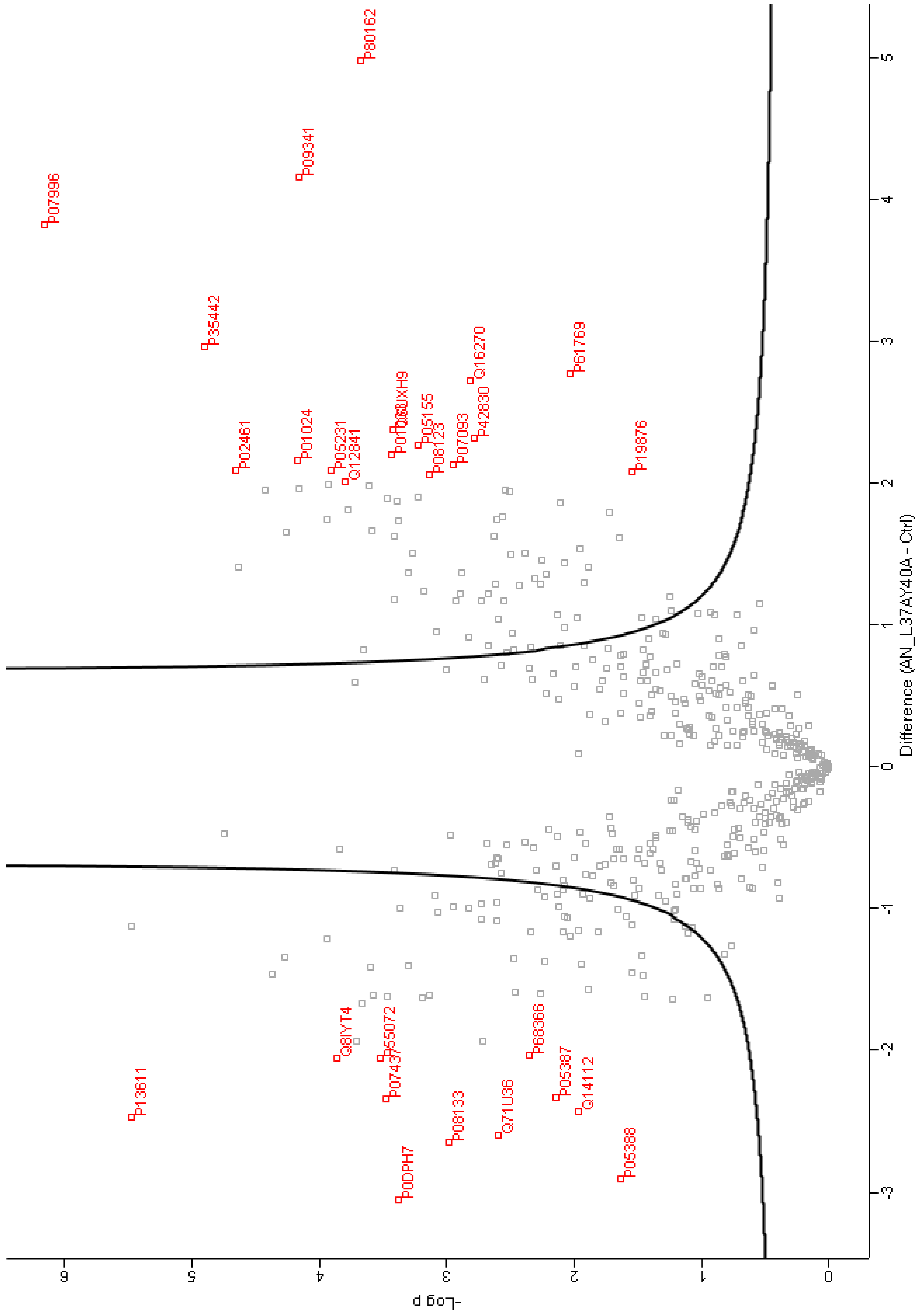
Difference (AN_L37AY40A - Ctrl)
-Log p
P80162
P09341
P07996
P35442
Q16270
P61769
P02461
P01024
P05231
Q12841
P05155
P08123
P07093
P42830
P19876
Q8IYT4
P55072
P07437
P68366
P05387
Q14112
P13611
Q71U36
P08133
P05388
P0DPH7

Figure S10. Dose-dependent increase in release of interleukin 6 from cells exposed to L37AY40A AN. Cells at an initial density of 2×10^5 cells per well in 6-well plates were cultured in basal media overnight, and then incubated in basal media containing either 15 or 30 µM wt or L37AY40A AN for 24 h. Culture supernatants were loaded on to 3–8% Tris-acetate SDS-PAGE gels under reducing condition, then electroblotted onto a PVDF membrane and probed with IL-6 Ab (6672). Lane labeled C represent control samples from cells incubated without AN. The full image of the blot is presented in Supplementary Figure S16. Densitometric analysis of the bands with optical density expressed as % compared to the sample incubated with 30 µM L37AY40A AN. Data are presented as means ± SD from three independent experiments. # indicates significant difference between the two concentrations of L37AY40A AN, at the $p < 0.05$ level. 'ns' indicates an absence of significant difference.

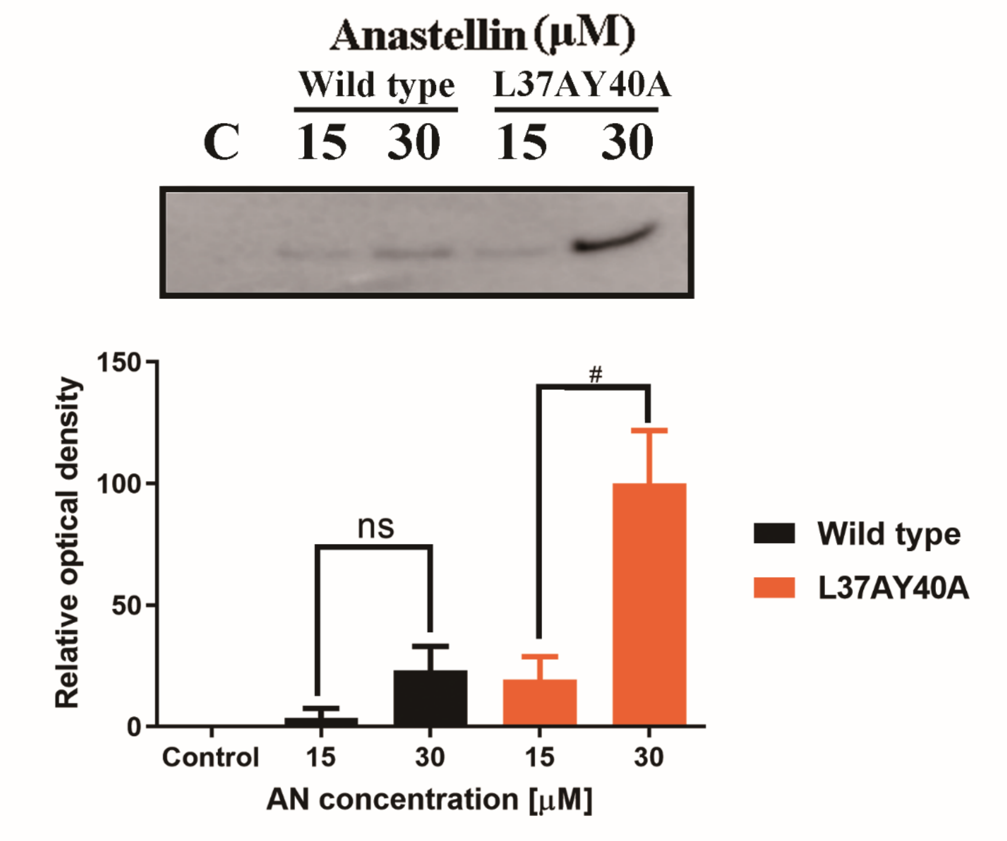

Figure S11. AN influences the cell adhesiveness of FN. Panel a: wt or L37AY40A AN (0.25 μM) was mixed with FN (1 μg mL^{-1}) in solution overnight at 21 °C and subsequently added to 96-well plates. HCASMCs (initial density 0.5 × 10^{4} cells per well) pre-loaded with calcein-AM were then added, and adherent cells quantified by fluorescence with λ_{ex} 490 nm and λ_{em} 520 nm. **Panel b**: As panel A except that 0.25 μM AN (wt or L37AY40A) was added to plasma FN (1 μg mL^{-1}) pre-coated on the plate, then incubated overnight before addition of dye-loaded cells. Columns labelled 'FN' represent control samples with 1 μg mL^{-1} FN but no added AN. Columns labelled 'wild type' and 'L37AY40A' represent control samples with AN but no FN. Data are presented as means ± SD from three independent experiments, analyzed by 2-way ANOVA with Tukey's multiple comparison test. * indicates significant difference from the control at the $p < 0.05$ level. # indicates significant difference between the two AN forms at the $p < 0.05$ level. 'ns' indicates as absence of significant differences between the two AN forms.

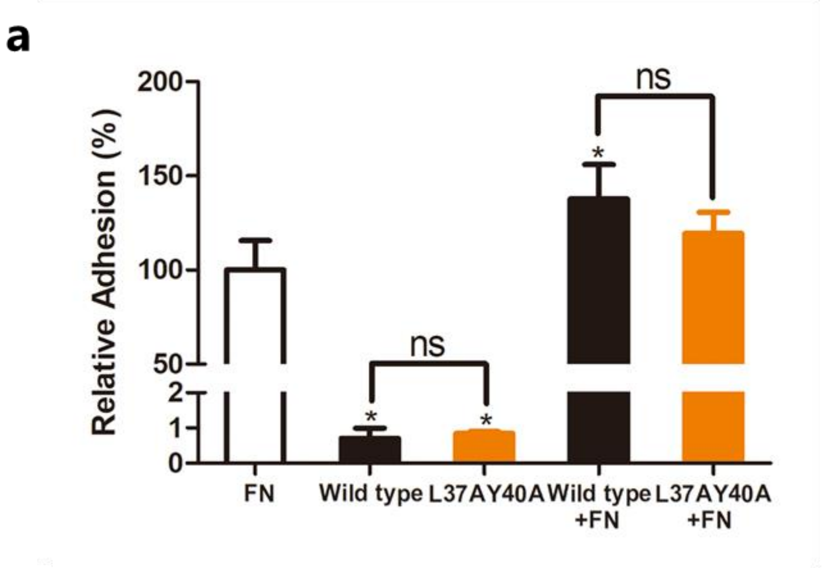

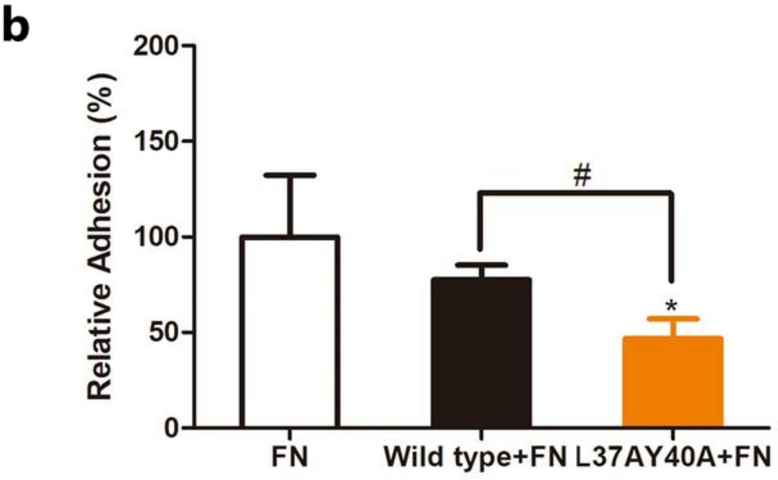

Figure S12. Wt AN, but not L37AY40A AN, induces FN polymerization. Panel a: The time course of FN aggregation was examined by turbidity measurements at 550 nm after mixing FN (0.4 mg mL^{-1}; 1.4 μM) with wt (2.5—40 μM, as indicated) or L37AY40A (40 μM) AN in 10 mM phosphate buffer, pH 7.4. **Panel b**: Determination of FN aggregates by sedimentation assays, where insoluble superfibronectin is separated from soluble material by centrifugation following overnight incubation of FN (1 μM) with wt or L37AY40A AN (40 μM) in 10 mM sodium phosphate buffer (pH 7.4) containing 5 mM EDTA and subsequent analysis by SDS-PAGE (see Materials and methods). Molecular mass markers are shown for reference. P = pellet, S = supernatant.

Figure S12

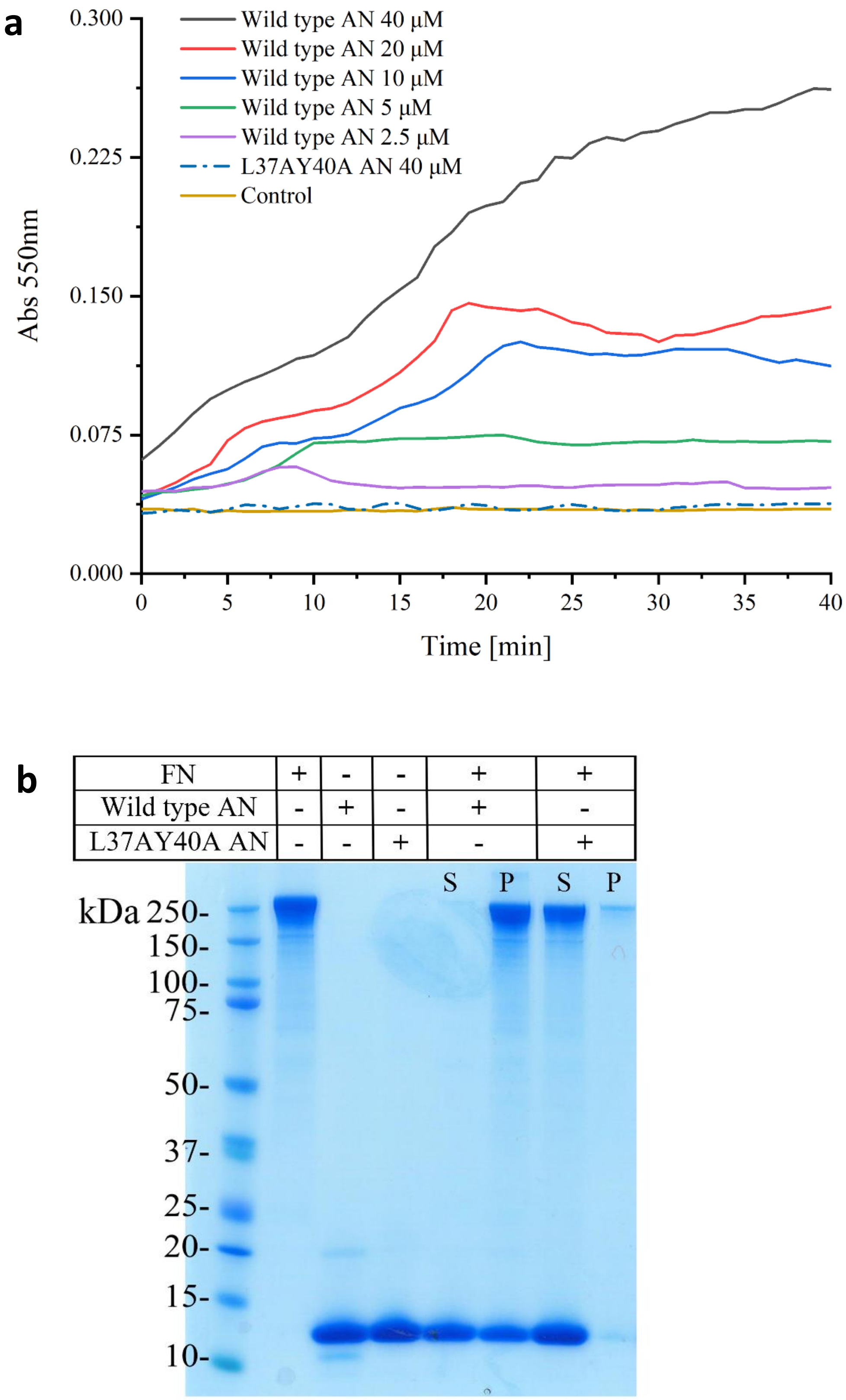
a
0.300
0.225
0.150
0.075
0.000
Abs 550nm
Wild type AN 40 μM
Wild type AN 20 μM
Wild type AN 10 μM
Wild type AN 5 μM
Wild type AN 2.5 μM
L37AY40A AN 40 μM
Control
0
5
10
15
20
25
30
35
40
Time [min]
b
FN + - - + +
Wild type AN - + - + -
L37AY40A AN - - + - +
S P S P
kDa 250-
150-
100-
75-
50-
37-
25-
20-
15-
10-

Figure S13. L37AY40A AN retains the ability of wt AN to bind to plasma FN precoated on plates. Plasma FN (20 µg mL^{-1}) in 10 mM phosphate buffer was pre-coated on plates then incubated with wt or L37AY40A AN overnight. The samples were then probed using an anti His-tag mAb antibody (1:500). Data are presented as means ± SD from three independent experiments, expressed as a % of values detected with the highest AN concentration (20 µM), and analyzed by 2-way ANOVA with Tukey's multiple comparison test. * indicates statistical significance when compared to the highest AN concentration at the $p < 0.05$ level. # indicates statistical differences between wt and L37AY40A AN at identical concentrations at the $p < 0.05$ level. 'ns' indicates an absence of statistical difference.

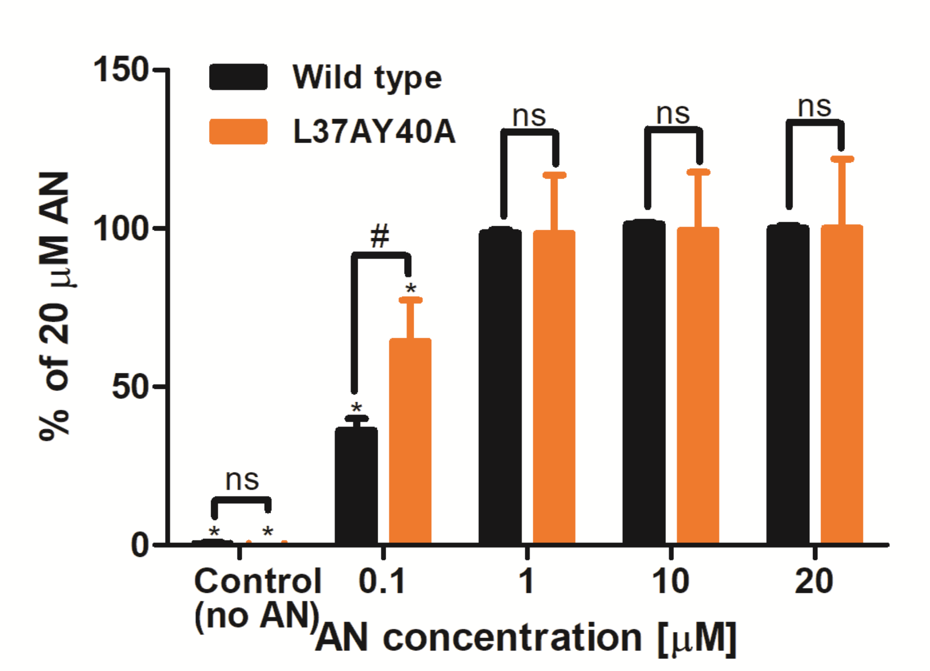

Figure S14. Heparin binding assay for FN exposed to wt or L37AY40A AN. **Panel a:** plasma FN (1 μg mL^{-1}) was incubated with wt or L37AY40A AN (0.25 μM) in 96-well plates overnight. Subsequently, 4 mg mL^{-1} of fluorescein-tagged heparin was added and incubated overnight, washed, and fluorescence detected using λex 495 nm and λem 525 nm. **Panel b:** plasma fibronectin (20 μg mL^{-1}) was precoated in plate overnight, then incubated with 1 μM wt or L37AY40A AN overnight before treatment with fluorescein-tagged heparin and assessment of bound heparin as described above. 'FN' represents controls with FN only, 'wild type' and 'L37AY40A' represent controls with AN only. Data are presented as means ± SD from three independent experiments, analyzed by 2-way ANOVA with Tukey's multiple comparison test. * Indicates significant difference from the control without AN at the $p < 0.05$ level. # Indicates significant differences from control without FN at the $p < 0.05$ level. 'ns' indicates an absence of significant differences.

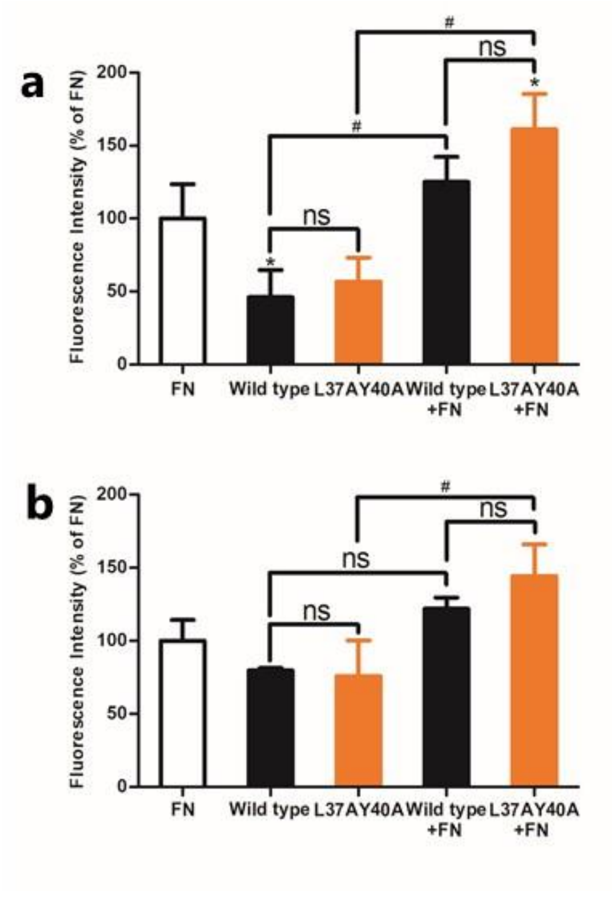

Figure S15. Translated sequence of L37AY40A AN. The mutated alanine residues corresponding to L37 and Y40 are underlined and highlighted in red. Residue numbering refer to the sequence of AN as defined in the UniProt entry for fibronectin (P02751).

MGNAPQPSHISKYILRWRPKNSVGRWKEATIPGH**A**NS**A**TIKG
LKPGVVYEGQLISIQQYGHQEVTRFDFTTTSTSTPHHHHHH

Figure S16. Full images of the blots probed with anti-pAb 2413 (panel a, cf Fig. S6), anti-β actin mAb (panel b, cf Fig S6), and anti-IL-6 Ab 6672 (panel c, cf Fig. S10).

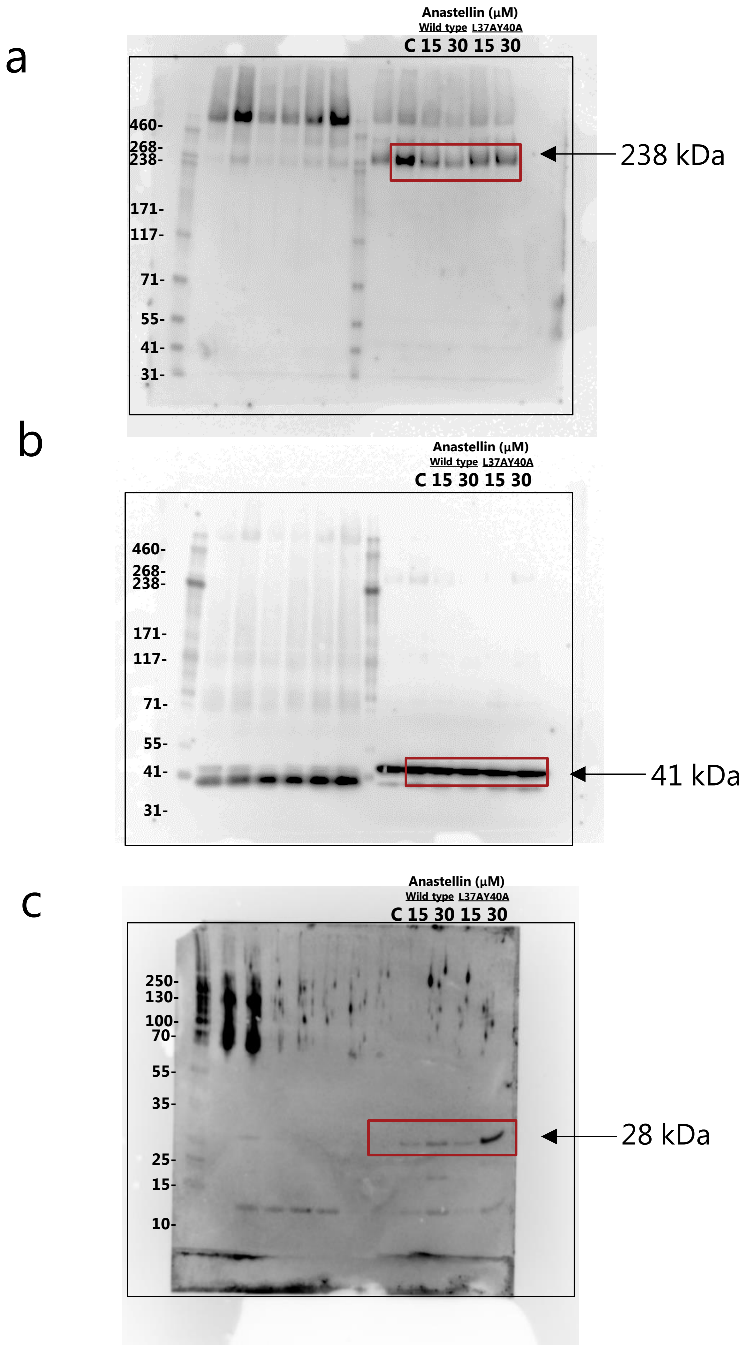

Supplement: Supplementary file 3 — Supplementary Information 3. [file 41598_2022_26359_MOESM3_ESM.pdf]
